# Relevance of Immune Infiltration and Clinical Outcomes in Pancreatic Ductal Adenocarcinoma Subtypes

**DOI:** 10.3389/fonc.2020.575264

**Published:** 2021-01-06

**Authors:** Rong Liu, Ya-Zhou Liao, Wei Zhang, Hong-Hao Zhou

**Affiliations:** ^1^Department of Clinical Pharmacology, Xiangya Hospital, Central South University, Changsha, China; ^2^Institute of Clinical Pharmacology, Central South University, Hunan Key Laboratory of Pharmacogenetics, Changsha, China; ^3^Engineering Research Center of Applied Technology of Pharmacogenomics, Ministry of Education, Changsha, China; ^4^National Clinical Research Center for Geriatric Disorders, Changsha, China; ^5^Department of Oral and Maxillofacial Surgery, Center of Stomatology, Xiangya Hospital, Central South University, Changsha, China

**Keywords:** tumor-immune infiltration, pancreatic ductal adenocarcinoma, overall survival, relapse free survival, M0 macrophages

## Abstract

**Purpose:**

Pancreatic ductal adenocarcinoma (PDAC) is a lethal cancer with high heterogeneity and dismal survival rates. Tumor immune microenvironment plays a critical role in sensitive to chemotherapy and prognosis. Herein, we determined the relevance of the composition of tumor-infiltrating immune cells to clinical outcomes in PDACs, and we evaluated these effects by molecular subtype.

**Experimental Design:**

Data of 1,274 samples from publically available datasets were collected. Molecular subtypes were predicted with support vector machine. Twenty-two subsets of immune cells were estimated with CIBERSORTx. The associations between each cell subset and overall survival (OS), relapse free survival (RFS), and complete response (CR) to chemotherapy were evaluated, modelling cellular proportions as quartiles.

**Results:**

An immune-related cluster was identified with unsupervised hierarchical clustering of hallmark pathways. Of the immune cells investigated, M0 macrophages emerged as closely associated with worse OS (HR =1.23, 95% CI = 1.15–1.31, p=1.57×10^-9^) and RFS (HR = 1.14, 95% CI =1.04–1.25, p=2.93×10^-3^), regardless of molecular subtypes. The CD8+ T cells conferred favorable survival. The neutrophils conferred poor OS overall (HR=1.17, 95% CI=1.10–1.23, p=1.74×10^-7^) and within the classical subtype. In the basal-like subtype, activated mast cells were associated with worse OS. Consensus clustering revealed six immune subgroups with distinct survival patterns and CR rates. The higher expression of PD1 was associated with better OS.

**Conclusions:**

The immune cellular composition infiltrate in PDAC are likely to have effects on prognosis. Further exploration of the cellular immune response has the potential to identify candidates for immunotherapy.

## Introduction

Pancreatic ductal adenocarcinoma (PDAC) constitutes the majority of pancreatic cancers, with the 5-year overall survival (OS) rate of only 9% (1). Most patients will succumb to the disease within a year after diagnosis with stage IV. PDAC is characterized by uniformly aggressive and biological diversity, and there is extensive heterogeneity among patients with respect to prognosis and treatment response. For example, among patients with stage I or II PDACs, some progress to stage IV within 4 weeks while in others there is relapse-free survival (RFS) longer than a year and a minority of patients are cured (2). Increasing molecular subtypes of PDAC have defined intertumoral heterogeneity at the genome changes in cancer cells level and transcriptome levels (3–6), which can identify prognosis subgroups in resectable tumor. Owning to the treatment-refectory nature, effective therapy for PDAC is limited (7). Currently, the FOLFIRINOX chemotherapy regimens (combination of gemcitabine with irinotecan, oxaliplatin, leucovorin, and 5-fluorouracil) can lower the death rate but increase toxicity and can be costly (8). Despite advancements in therapeutic regimen modalities, the prognosis of PDACs achieved incremental improvements. Thus, there is urgent need to explore novel therapeutic strategies for PDAC.

The tumor microenvironment of PDAC comprises the stroma in addition to cancer cells. The stroma includes a variety of components, and infiltrating immune cells that comprise about half of the cellular PDAC have the potential to be druggable for improved clinical outcome. Emerging evidence has shown the important role of tumor infiltrating immune cells, such as dendritic and macrophages (9), regulatory T and cytotoxic T cells, in the tumorigenesis, development, progression, and distant metastasis of tumor, and also its resistivity to treatment. The factions of CD8+ T cells and macrophages infiltrated in the tumor microenvironment have impact on prognosis in a variety of malignancies, such as melanoma and breast cancers (10, 11). Immune checkpoint inhibitor (ICI) therapy, which can strengthen the antitumor response, has made impressive improvements in the treatment of solid tumors such as melanoma (12), gastric cancer (13), or haematological malignancies (14). However, a dismal response to ICI therapy by PDAC in comparison with other solid tumors (12, 15, 16) may accounting for merely 4% of tumor cells with the expression of PD-L1 (17); most of PDAC tumors present intermediate to high expression of infiltrating T cells that are mainly CD4+ T cells rather than CD8+ T cells (17) and the relatively low mutational burden (18). Great efforts are put into the improvement of the effect of immune checkpoint inhibitor therapy (19, 20). And it is necessary to identify novel therapeutic targets for immunotherapy of PDAC. It has been reported that resistance to immunotherapy in PDAC might account for the imbalance between immunosuppressive and effector immune cops in the tumor microenvironment (21). Xu et al. reported that the proportions of activated dendritic cells and M0 macrophages in tumor tissues were significantly greater than that in adjacent tissues and independent prognosis factors (22). Mahajan and colleagues reported that immune cells might affect the composition of the pancreatic stroma to be associated with progression-free survival of PDAC (23). However, since PDAC is a highly heterogeneous disease, they didn’t determine whether these effects differ by molecular subtype. Thus, it is necessary to conduct a pooled analysis with large-scale datasets covering the molecular diversity of PDAC.

Herein, the immune landscape of PDAC and the associations between immune cellular subtypes, immunotherapy targets, and OS, RFS and complete response (CR) to chemotherapy overall and by molecular subtypes were comprehensively investigated. A computational framework CIBERSORTx (24), which enables removed batch effect from different platforms, was to accurately estimate cell type fractions from mRNA profiles of intact tissues on the basis of mixed cellular gene expression data in 1,274 PDAC patients with clinical outcomes from 9 datasets. A profound understanding was gained on the diverse association between clinical outcomes and different functional immune cell subsets within different molecular subtypes.

## Materials and Methods

### Study Population

**Figure 1:**
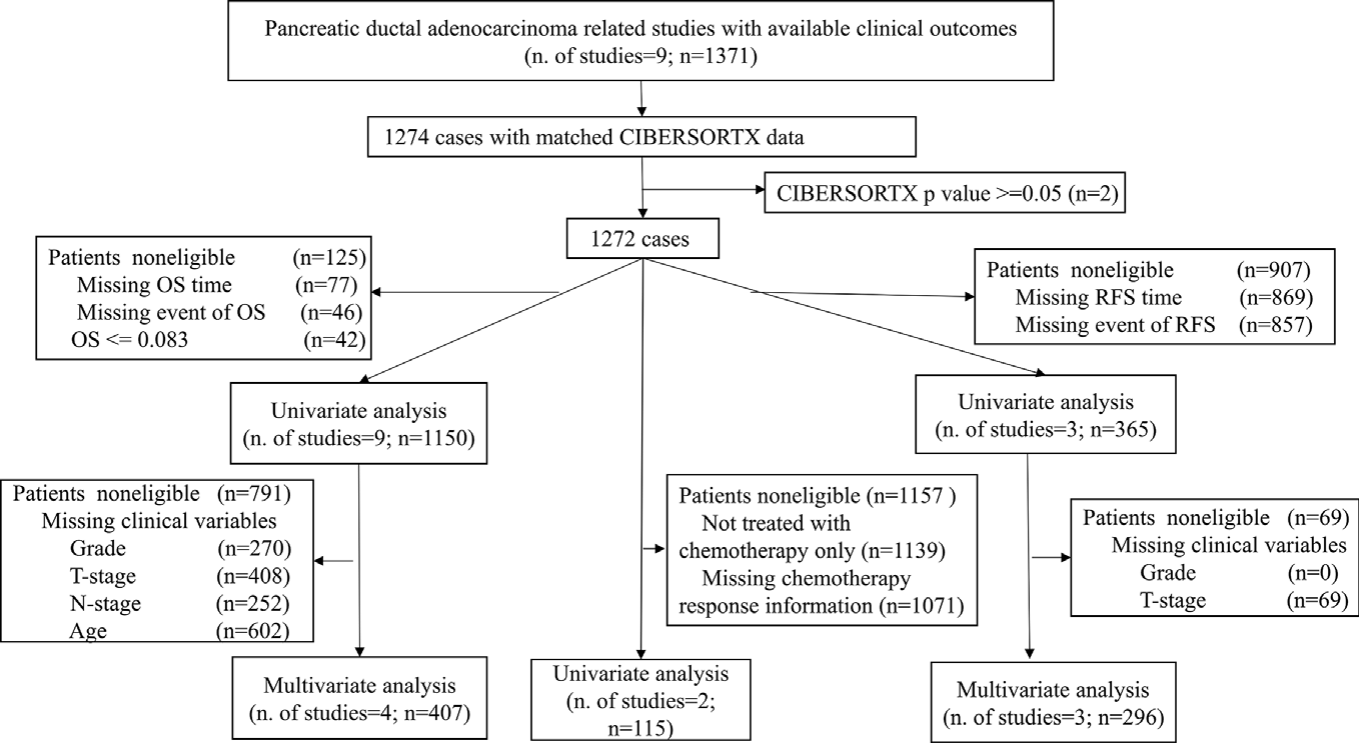
Flowchart of this study. This picture illustrates the flow of samples at each phase of statistical analysis. PDAC, Pancreatic ductal adenocarcinoma; OS, Overall survival; RFS, Relapse-free survival.

This study took advantage of publicly available databases. To identify genome-wide gene expression dataset of PDACs, the International Cancer Genome Consortium (ICGC, https://icgc.org/), The Cancer Genome Atlas (TCGA, https://portal.gdc.cancer.gov/), the ArrayExpress (https://www.ebi.ac.uk/arrayexpress/), and Gene Expression Omnibus (GEO, https://www.ncbi.nlm.nih.gov/geo/) were systematically searched with keywords “pancreatic cancer“ or “pancreatic carcinoma.” Tumor histology other than ductal adenocarcinoma was excluded. In total, 1,274 patients from 9 studies (**Table S1**) with matched mRNA expression dataset and clinical outcomes were collected. **Figure 1** illustrates which samples were used at each phase of analysis.

### Data Processing

Normalized RNA-seq data (fragments per kilobase of transcript per million [FPKM] values, GRCh37) for PAAD-AU and PACA-CA samples were downloaded from the ICGC data portal (dcc.icgc.org/) on March 30, 2020 (release 28). Only samples from primary tumor were included. Normalized RNA-seq data (FPKM values) from TCGA were downloaded from the GDC browser. FPKM values were transformed into log2(transcripts per kilobase million[TPM] values +1), which are more similar to data from microarrays (25).

For datasets from GEO and ArrayExpress, the normalized expression data and relevant clinicopathological data and clinical outcomes in series matrix format were derived. We searched the supplements of the original publications when the clinical information was not listed in the series matrix files. For each sample, probes that can’t be annotated with a specific gene were filtered. The mean value of all probes mapped to the gene was computed when multiple probes correspond to a single gene.

### PDAC Transcriptomic Molecular Subtype

Samples’ molecular subtypes were determined according to the expression levels of gene signatures described in Bailey (3), Collisson (4), and Moffitt (5) using support vector machine (SVM) algorithms. Collisson subtype of GSE17891 and Moffitt subtype of GSE71729 and E-MTAB-6134 were obtained from the original publications. Bailey subtyping was based on genes from differential expression analysis between subtypes results from the original publication (3), which were filtered for genes with an adjusted p < 0.005 and log2 fold change>2, resulting in 87 ADEX, 118 squamous, 18 progenitor, and 111 immunogenic specific genes. Collisson and Moffitt subtyping were according to the genes belonging to the 62 and 50 gene signature in the original publications (4, 5), respectively. Gene expressions were first scaled so that the 2.5% and 97.5% quantiles equalled -1 and +1 by sample within each dataset. Predictive models based on scaled expression and subtypes of Bailey, Collisson, and Moffitt were trained using SVM. Then, the models were applied for all samples in the resting datasets. For robustness, the SVM model development and predict process were repeated 1,000 times, then samples were assigned subtypes with average predictive probability larger than 0.7. The SVM models were developed using the e1071 package in R. The distributions of different molecular subtypes and clinical characteristics for every dataset are shown in **Table S2**.

### Pathways, Immune Content Score, and Infiltrating Immune Cell Subsets

The gene lists of hallmark pathways were obtained from the Molecular Signatures Database (26), and those unrelated to PDAC were removed (myogenesis, protein secretion, UV response, heme metabolism, coagulation, peroxisomes, allograft rejection, spermatogenesis, androgen response, estrogen response, and rationale for exclusion were listed in **Table S3**). Pathway scores were computed as the average gene expression within each pathway. For heatmap, pathway scores were first scaled so that the 2.5% and 97.5% quantiles equalled -1 and +1 by sample within each dataset and then by pathway. The immune content score was calculated as the mean level of 265 immune cell specific genes from publication (27). To estimate proportion of different immune cell subsets, CIBERSORTx (24) (https://cibersortx.stanford.edu/), which is designed for enumerating cell composition from mixed tissue with functionalities for batch correction using cross-platform data normalization, was utilized. Both the relative and absolute immune cell proportion from each normalized gene expression dataset (permutation number=1000, B-mode batch correction and disable quantile normalization for RNA-Seq data as recommended) were computed with LM22 gene signature as reference. A measurement of the reliability of the deconvolution results (p-value) was calculated for each sample.

### Immune Checkpoint Molecules

The associations between immune checkpoint molecules and prognosis of PDACs were investigated. We divided PD-1 gene expression by the immune content score because PD-1 is identified to be specifically expressed in immune cells (28). CTLA-4 is closely specific to T-cells (28), so that the expression values of CTLA-4 were divided by the T-cell abundance (the total relative proportions of T-cells estimated from CIBERSORTx multiplied by immune content score). Since not an immune cell-specific gene, the expression of PD-L1 and PD-L2 were not adjusted.

### Statistical Analyses

Clinical information on age, tumor size, nodal, metastasis, grade, response to chemotherapy, and survival was collected. The clinical endpoints were RFS and OS. In dataset E-MTAB-6134, disease-free survival (DFS) rather than RFS was provided, and then RFS is equal to DFS when DFS time is shorter than OS, which indicates that these is local or distance relapse before the patients’ death. For TCGA dataset, when response to chemotherapy is not provided, the response to chemotherapy drug (such as gemcitabine) is used. The types of clinical outcomes derived from each study can be found in **Table S1**.

To access the association between the factions of tumor infiltrating immune cells and survival, survival analysis was performed. In the association analysis, cell fractions with over 50% of the samples with 0% of proportion were excluded. Patients with a follow-up or OS time of shorter than 30 days that were assumed to have postoperative complications and those with a CIBERSORTx p-value ≥ 0.05 were filtered. Cox regression analysis was applied to time-to-event data, with quartiles of each of the 19 immune cells fractions modelled as continuous. Quartiles (25%, 50%, and 75%) were computed overall and by different molecular subtypes within each dataset. Study was included in the Cox regression models as strata variable. The log-rank test was utilized to test the differences in the survival rate between subtypes as illustrated in Kaplan-Meier survival curves. The associations between survival and age, grade, tumor size, nodal (negative, positive), and metastasis were evaluated. Ordinal categories of grade were modelled as continuous variables. The statistically significant immune cell subsets and covariates in the univariate analysis were selected in the multivariable Cox regression models. Furthermore, select variables from univariate cox regression analysis may lead to the exclusion of strong associated factors when confounding is properly controlled (29). To address this, multivariable Cox regression models were developed *via* the penalized maximum likelihood performed with the R package glmnet (30). According to the result of 1,000 cross-validation tests, the penalization factor was determined.

To evaluate the association between each immune cell subsets and CR to chemotherapy, logistic regression analysis was conducted with quartiles of fractions of each immune cell subsets treated as continuous variables.

Chi-square tests were performed to access the association between categorical variables. Pairwise correlation relationships between the relative fractions of immune cell subsets were evaluated with the Pearson’s correlation analysis, then illustrated in heatmaps. The associations between the immune checkpoints and prognosis were estimated with the statistical techniques described above.

In this study, all statistical analyses were conducted using the R version 3.6.2 (31). Results were thought to be significant when p-value<0.05.

### Consensus Clustering for Tumor-Infiltrating Immune Cells

To investigate tumor-infiltrating immune cells infiltration patterns of PDAC, consensus clustering analysis was performed to immune cell proportions in patients with a CIBERSORTx p<0.05. To make it comparable between the rare (low overall ratio) and the abundant (high overall ratio) cell subsets, the relative fraction values were scaled between 0 (the smallest value actually) and 1 (the largest value actually) for individual type of immune cell. To ensure the stability of classification, clustering was performed using Euclidean distance with k-means algorithm using the ConsensuClusterPlus R package (32) with 1,000 re-samplings. The association between clinical outcomes and tumor-infiltrating immune cell clusters was tested with the above-mentioned statistical approaches.

## Results

### Immune Landscape of PDAC

**Figure 2:**
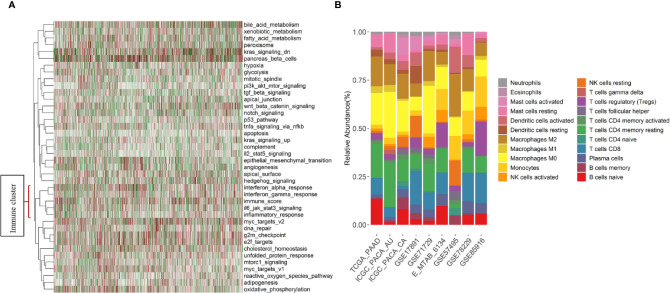
Immune pathways, immune content score, and summary of estimated fractions of 22 tumor-infiltrating immune cell subtypes by study. **(A)** Heatmap of the unsupervised hierarchical clustering based on hallmark pathways’ scores and the immune content score in 1,274 PDAC samples. Immune-related pathways and the immune content score formed a cluster that located at the bottom of the heatmap. **(B)** Stacked bar charts summarizing immune cell subtype fractions. NK cells, natural killer cells.

First, to explore underlying patterns, unsupervised hierarchical clustering of all 1,274 PDAC samples was conducted with the hallmark pathways (26) (**Figure 2A**). Interesting, we found that pathways related to immune response (interferon alpha, interferon gamma, IL6, and inflammatory response pathway) were strongly intercorrelated. In addition, the immune content score clustered with the above immune-related pathways and formed an immune cluster (**Figure 2A**). Second, the association between immune content score and clinical outcomes was investigated. No significant association exists between the immune content score and OS, RFS, CR, grade, nodal, tumor size, metastasis, and molecular subtypes (**Figure S1**).

### Molecular Subtypes and Association With Clinical Outcomes

SVM-based predictive models for Collisson, Bailey, and Moffitt molecular subtypes were developed, respectively. Survival analysis displayed significant differences in OS (log rank p =7.6×10^-16^, **Figure S2a**) and RFS (log rank p = 9.71×10^-11^, **Figure S2b**) among the Moffitt subtypes. The survival of PDAC patients within the classical subtype was better than that of basal-like subtypes, which was in accordance with the finding of Moffitt et al. (5). The ratios of CR to chemotherapy are 0.66 and 0.42 within basal-like and classical subtypes (p=0.06, **Figure S2c**). The Bailey and Collisson subtypes were concordantly associated with OS, RFS, and CR (**Figure S2d-i**).

### Evaluate Tumor-Immune Infiltration With CIBERSORTx

Individual immune cell subsets were deconvoluted from expression data of 1,274 samples and summarized (**Figure 2B**). The gene expression data of the 10 datasets included in this study were generated with seven different platforms (**Table S1**). The average proportion of represented genes included in the LM22 signature across datasets was 85.7% (**Figure S3**). The most and least variable immune cell subsets of the samples across datasets were the M0 macrophages [mean = 14.2%, standard deviation (SD) = 6.51%] and the neutrophils (mean = 0.95%, SD = 0.67%), respectively. Naïve CD4 T cells, T cells gamma delta, and eosinophils, with over 50% of the samples having no infiltration, were filtered before the association analysis was performed (**Figure S4**).

Pairwise correlations between the ratios of 22 subsets of PDAC tumor-infiltrating immune cell were weakly to moderately correlated overall (**Figure S5**) and in the classical (**Figure S6**) and basal-like subtypes (**Figure S7**). In general, monocytes and T cells regulatory (Tregs) represented the strongest positive correlation (R=0.59), whereas the M2 macrophages and Tregs showed the highest negative correlation (R=-0.48). Regardless of different Moffitt subtypes, the patterns of correlation between the fraction of 22 immune cell types infiltrated in PDAC were similar.

### Immune Cell Subsets Are Prognostic

**Figure 3:**
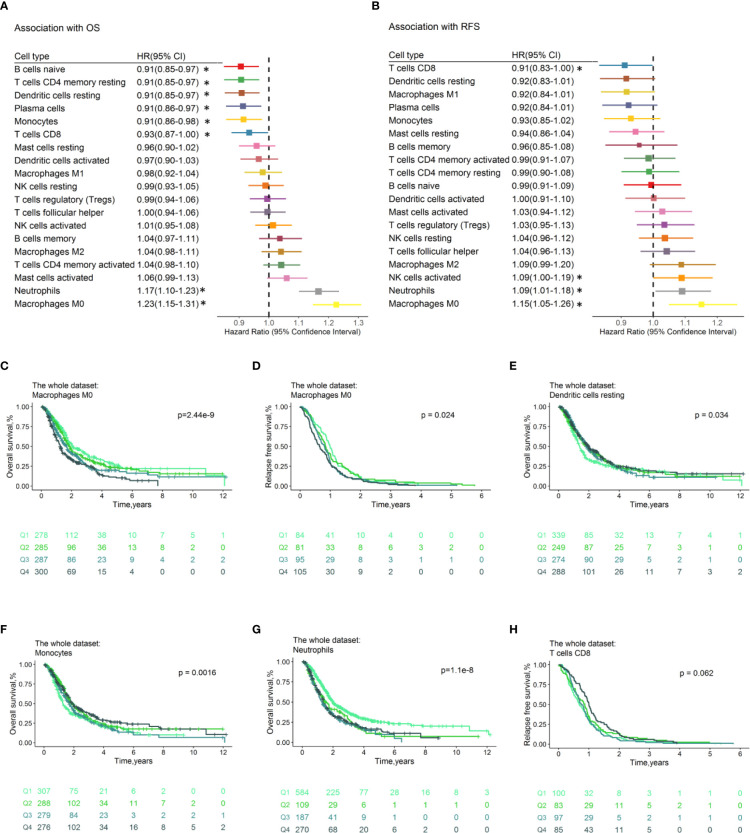
Prognostic associations of immune cell subsets overall. Unadjusted HRs (boxes) and corresponding 95% CIs (horizontal lines) for immune cell subtypes associated with OS **(A)** and RFS **(B)** are represented. Box size is inversely proportional to the standard error of HR. * indicates HRs with a p value < 0.05. Immune cell fractions are stratified as quartiles for the survival curves **(C–H)**, and p-values calculated using log-rank tests were drawn. HR, hazard ratio; CI, confidence interval; OS, Overall survival; RFS, Relapse-free survival.

The proportion of tumor-infiltrating immune cell subsets were associated with survival of PDACs. The samples with CIBERSORTx p ≥ 0.05 or OS ≤ 1 month were filtered, 1,150 PDAC cases were left with a median OS time of 1.89 years (740 events), and 365 cases have the RFS information available (median RFS time=0.83, all samples relapsed). Forest plots illustrated the HRs and 95% CIs for immune cell subsets for OS (**Figure 3A**) and RFS (**Figure 3B**). Generally, the M0 macrophages were associated with poor OS (HR=1.23, 95% CI=1.15–1.31; p= 1.57×10^-9^) and RFS (HR=1.15, 95% CI=1.05–1.26; p=2.93×10^-3^). Neutrophils conferred worse OS (HR=1.17, 95% CI=1.10–1.23; p=1.74×10^−7^) and RFS (HR=1.09, 95% CI=1.01–1.18; p=3.29×10^-2^). CD8 + T cells were associated with favorable OS (HR=0.93, 95% CI=0.87–1.00; p=4.19×10^-2^) and RFS (HR=0.91, 95% CI=0.83–1.00; p=4.52×10^-2^). Tumor-infiltrating Cytotoxic CD8+ T cell is found to be predictive for response to ICI therapy. A recent study suggested that CD8+ T cells have significant impact on survival of pancreatic cancer patients (33). In addition, the resting of dendritic cells (HR=0.91, 95% CI=0.85–0.97; p = 3.50×10^-3^), monocytes (HR=0.91, 95% CI =0.85–0.97; p=8.05×10^-3^), naïve B cells (HR=0.91, 95% CI =0.85–0.97; p=2.88×10^-3^), and plasma cells (HR=0.91, 95% CI =0.86–0.97; p=6.09×10^-3^) were associated with favorable OS. The HRs and corresponding 95% CI of the above significant immune cell subsets for OS in separate datasets was shown in **Figure S8**, indicating the reliability of our analysis. Multivariable Cox regression analyses adjusted for prognostic variables (age, tumor size, nodal, and grade; **Table S4a**) were performed, and results revealed that the neutrophils (HR =1.18, 95% CI = 1.05–1.32; p=5.18×10^-3^) and plasma cells (HR =0.88, 95% CI =0.77–0.99; p=4.11×10^-2^) make contributions to the model for OS (**Table S4b**). As for RFS, there is no immune cell subset left in the model (p<0.05) after adjusting for tumor size and grade (**Table S4c-4d**). In multivariable Cox regression models with penalized maximum likelihood estimation, the cell type with the highest HR point estimates in OS (HR=1.19; **Table S4e**) is M0 macrophage, and the cell type with the lowest HR point estimates in RFS (0.92; **Table S4f**) are plasma cells and resting CD4 memory T cells. We also conducted association analysis with the immune cell infiltration estimated in “absolute mode” of CIBERSORTx, and we found that the results are accordance with that of “relative mode” (**Figure S9**).

### Prognostic Effect of Tumor Infiltrating Immune Cells by Molecular Subtype

Exploratory analysis to evaluate the prognostic effect of immune cell types by Moffitt, Collisson, and Bailey subtypes were conducted. We noticed that these are consistent and variations regarding the prognostic effect of immune cells by molecular subtypes for OS and RFS.

**Figure 4:**
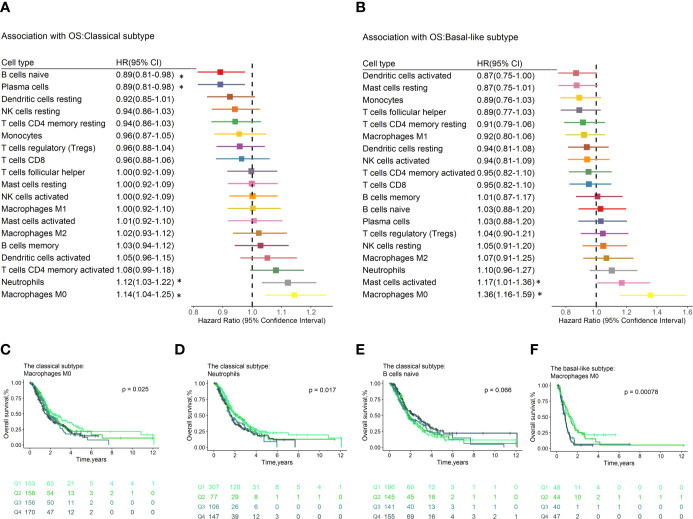
Forest plot depicted the results of the univariate Cox regression models of immune cell infiltration for OS by Moffitt molecular subtypes. Unadjusted HRs and 95% CIs for the association with OS by Moffitt subtypes **(A, B)** are represented with boxes and horizontal lines, respectively. The size of the box is inversely proportional to the standard error of HR. * indicates a p-value < 0.05. Immune cell fractions are stratified as quartiles for the survival curves **(C–F)**, and p-values calculated using log-rank tests were drawn. HR, hazard ratio; OS, overall survival; CI, confidence interval.

In the classical subtype defined by Moffitt (**Figure 4A**), high levels of M0 macrophages conferred poor OS (HR=1.14, 95% CI=1.04–1.25; p= 3.64×10^−3^, **Figure 4C**) and RFS (HR=1.16, 95% CI=1.01–1.33, p=4.12×10^-2^, **Figure S10c**), while plasma cells were associated with superior OS (HR=0.89, 95% CI=0.81–0.98, p=1.25×10^-2^) and RFS (HR=0.80, 95% CI=0.68–0.93, p=3.54×10^-3^, **Figure S10d**). In addition, neutrophils (HR=1.12, 95% CI=1.03–1.22, p=6.50×10^-3^, **Figure 4D**) conferred worse OS. The naïve B cells (HR=0.89, 95% CI=0.81–0.98, p=1.27×10^-2^, **Figure 4E**) were associated with favorable OS. The M0 macrophages (HR = 1.27, 95% CI = 1.08–1.49; p = 3.13×10^-3^) and neutrophils (HR = 1.19, 95% CI =1.03–1.36; p = 1.52×10^-2^) still significantly associated with OS (**Table S4g**) after adjusting for known clinical prognosis factor in multivariate Cox regression model, whereas the plasma cells (HR=0.78, 95% CI=0.65–0.94, p=8.53×10^-3^) contributed to the multivariate model for RFS (**Table S4h**). In the multivariable Cox proportional hazard models with penalized maximum likelihood estimation, the M0 macrophages had the highest point estimate of HR in OS (HR=1.13) (**Table S4i**), and plasma cells were associated with RFS (HR=0.96, **Table S4j**).

In the basal-like subpopulation (**Figure 4B**), the M0 Macrophages (HR = 1.36, 95% CI = 1.16–1.59; p = 1.70×10^-4^, **Figure 4F**) and the activated mast cells (HR = 1.17, 95% CI = 1.01–1.36; p = 4.07×10^-2^) showed association with poor OS. Multivariable analyses showed that no immune cell subset contributed to the model for OS (**Table S4k**), and the M2 macrophages (HR = 1.68, 95% CI = 1.08–2.62; p = 2.24×10^-2^) contributed to the model for RFS (**Table S4l**). No immune cell subset contributes to models for OS and RFS in multivariable Cox proportional hazard models with penalized maximum likelihood estimation.

Among subgroups defined by Bailey et al., the high proportions of M0 Macrophages conferred worse OS in ADEX and pancreatic progenitor subtypes. Monocytes were associated with superior OS and RFS within pancreatic progenitor subtypes. Neutrophils and M1 macrophages were associated with poor OS (**Figure S11**) and good RFS (**Figure S12**) within ADEX subtype, respectively. Within Collisson subtype, the neutrophils and M0 macrophages were associated with poor survival in classical PDA subtype but not others (**Figure S13**). These findings warrant cautious interpretation as, although as a relatively large combined cohort, they are relatively underpowered given the small sample size within certain subgroups.

In addition, the prognostic effect of immune cell types by grade (**Figure S14** and **Figure S15**) and nodal status (**Figure S16**) were also explored. Overall, the high level of M0 macrophages showed association with poor OS and RFS regardless of grade and status.

### Prognosis Effect of Immune Cells of CR to Chemotherapy

Data from TCGA and ICGC_CA were utilized to assess the relevance between immune cell fractions and CR to chemotherapy (**Figure S17**). Patients who achieved CR were likely to have improved survival (34). Overall, activated dendritic cells (odds ratio [OR]=0.85, 95% CI 0.60–1.19; p =3.44×10^-1^, **Figure S17b**) showed the lowest OR, suggesting association with a low ratio of CR. Monocytes showed the strongest association with CR (OR=1.23, 95% CI 0.94–1.84; p=2.33×10^-1^, **Figure S17c**), although there was no association between immune cell subtypes and CR statistically significance that might account for small sample size. These might somewhat interpret the association between monocytes and favorable OS. Since none of the clinical covariates showed association with CR (**Table S4n**) in univariate analysis, multivariable models were not developed. Analyses conducted separately by Moffitt subtypes (**Figure S18**) showed no significant results.

### Immune Clusters Associated With Clinical Outcomes and Molecular Subtypes

**Figure 5:**
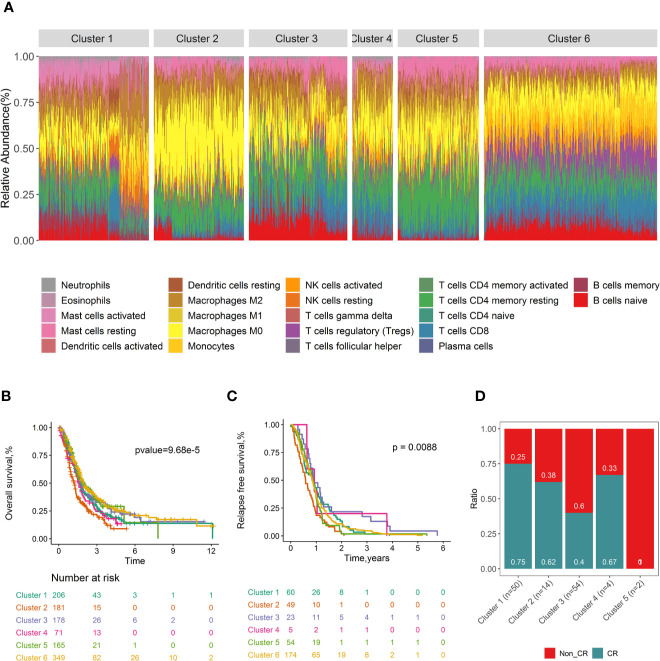
Consensus clustering of all 1,274 PDAC samples according to immune cell fractions and survival curves by immune clusters. Stacked bar charts of samples ordered by immune cluster assignment **(A)**. Survival plots by immune cluster separately for OS **(B)** and RFS **(C)**. Spine plots illustrating the distribution of CR rates within quartiles of tumour infiltrating immune cell subtypes **(D)**. P-values from log-rank tests are shown. OS, Overall survival; RFS, Relapse-free survival; CR, complete response.

Consensus clustering (32) was conducted to investigate whether distinguishing patterns of tumor-infiltrating immune cell can be revealed according to the tumor infiltrates immune cell types of PDACs. Six clusters were determined based on the cumulative distribution function, the proportion of ambiguous clusters values, the consensus matrix, and consensus cluster index (**Figure S19**). The tumor immune cell infiltration ratios by these six clusters are depicted in **Figure 5A**, and the distributions of these immune subtypes were shown in boxplots (**Figure S20**). Distinct patterns of OS (log rank p =9.68×10^-5^, **Figure 5B**), RFS (log rank p=8.80×10^-3^, **Figure 5C**), and CR (p value= 4.50×10^-3^, **Figure 5D**) were exhibited between immune clusters. Cluster 3, with high proportions of naïve B cells and low level of M0 macrophages, was conferred improved OS and RFS. Conversely, cluster 2, with high levels of M0 macrophages and low levels of naïve B cells, was associated with poor OS. Immune clusters were associated with tumor stage (p= 3.41×10^-8^, **Figure S21a**), tumor metastasis (p= 2.52×10^-13^, **Figure S21b**), and grade (p<2.2×10^-16^, **Figure S21c**). No significant association was found between the immune cluster and tumor nodal (p= 5.02×10^-1^, **Figure S21d**) and patient’s age (p= 4.81×10^-1^, **Figure S21e**).

The distribution of immune clusters was different within the Moffitt subtype (p value=7.50×10^−6^, **Figure S21f**), and this relationship was mainly accounting for the enrichment of the classical subtype (90.2% to 77.5% overall) in cluster 5. The immune clusters were also associated with Collisson molecular subtype (p value < 2.20 ×10^−16^, **Figure S21g**) and Bailey molecular subtype (p value < 2.20 ×10^−16^, **Figure S21h**). In general, these results indicated that variability existed in the nature of the immune cells infiltrated in PDAC tumours, to some extent attributed to molecular characteristics of the tumor. This may have impact on the clinical outcomes of patients.

### Immune Checkpoints Are Prognostic

**Figure 6:**
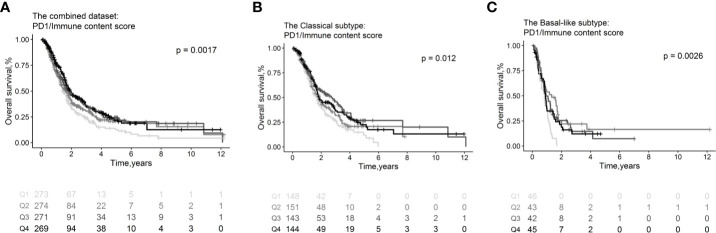
Survival plots illustrating prognosis associations of immune checkpoint molecules. Associations of PD1 with OS in the combined whole cohort with 1,150 PDAC patients **(A)**, within the classical **(B)** and basal-like **(C)** subtypes. In the survival curves, immune cell subsets are stratified as quartiles. P-values from log-rank tests are depicted. OS, Overall survival.

Immune checkpoint molecules, which be targeted by drugs, were tested in association with clinical outcomes of PDAC patients (**Table S5**). We found that high expression levels of PD-1 normalized to the estimated immune content score conferred favorable OS (HR=0.88, 95% CI=0.82–0.94; p= 3.35×10^−4^, **Figure 6A**) overall and in the classical (HR=0.88, 95% CI=0.80–0.97; p=1.11×10^−2^, **Figure 6B**) and basal-like subtypes (HR=0.88, 95% CI =0.74–1.03; p= 1.09×10^−1^, **Figure 6C**). The PD-1 receptor interacts with ligands PD-L1 and PD-L2. We found that PDL-1 (HR=1.04, 95% CI = 0.98–1.11; p =1.99×10^−1^) and PDL-2 (HR=1.05, 95% CI = 0.98–1.12; p = 1.70×10^−1^) were correlated with worse OS overall; however, the associations were not statistically significant. Meanwhile, there are no immune checkpoint molecules with significant association relationship with RFS (**Table S5b**) or CR (**Table S5c**).

## Discussion

It is a complex network of the interaction between the tumor-infiltrating lymphocytes and its antitumor effects against PDAC. In this study, using data from 1,274 samples, an immune-pathway cluster was demonstrated. The fractions of 22 immune cell types were deconvoluted from genome-wide gene expression data generated from bulk tumor tissue, and the associations between immune cell types and clinical outcomes were evaluated. In line with the limited literature about PDAC, the negative association between M0 macrophages and neutrophils and poor survival were validated (22, 35). Meanwhile, CD8+ T cells confer better favorable prognosis (36, 37). The PD-1 is associated with favorable survival. Differences exist in tumor immune infiltration in PDACs and may be important determinants of clinical outcomes within molecular subtypes. Our findings are consistent with those of the literature, which increases confidence that computational evaluation is a reasonable method to estimate tumor-infiltrating lymphocytes fractions.

Tissues are complicated ecosystems that include multiple cell types, while exploring tissue composition is still challenging. Limited cell types can be simultaneously interrogated with traditional immunophenotyping techniques, such as flow cytometry and immunohistochemistry, which are based on small combinations of preselected marker genes. Single-cell RNA sequencing, a newly developed method, makes transcriptional profiling of thousands of individual cells available. However, analyses of large samples (especially fixed clinical specimens such as formalin-fixed, paraffin embedded samples) are not yet practical. Computational techniques for the assessment of cellular heterogeneity from gene expression profiles of mixture samples were established over the past decade, such as CIBERSORT (38), xCell (39), and TRUST (40). Recently, the extended version of CIBERSORT, namely CIBERSORTx (24), was established. This framework can accurately infer cell type abundance from RNA profiles of tissues with newly developed functions for cross-platform data normalization. Since the datasets utilized in this study generated with both microarray and RNA-seq techniques, CIBERSORTx was selected by us and the “B-mode” batch normalization functions were chosen for RNA-seq data.

Overall, of the cell subtypes investigated, as the most frequent infiltrated immune cells, high proportions of M0 macrophages conferred worse prognosis, regardless of OS, RFS, and CR in the combined datasets and among different molecular subtypes. Macrophages are protumoral and immunosuppressive *via* simulating tumor proliferation, angiogenesis and metastasis, and associated with poor prognosis in solid tumors (9). Tumor-associated macrophages can suppress responses to cancer therapeutics. The M0 states of macrophages can be polarized into M1 and M2 status, which have distinct immunoregulatory functions, namely, antitumoral and protumoral, respectively. In addition, the progression of PDAC can be promoted by tumor associated macrophages by promoting the Warburg effect through CCL18/NF-kB/VCAM-1 pathway (41). Herein, the prognosis effect of M0 macrophages was highlighted. However, the mechanism of how the fraction of M0 macrophages affect sensitivity to chemotherapy and progress of PDACs still need further experiment exploration. The fraction of CD8+ T cells are associated with good OS and RFS. CD8+ T cells play critical roles in protecting immunity against intracellular pathogens and tumors (42). Tumor-infiltrating cytotoxic CD8+ T cell is found to be predictive for response to immune checkpoint inhibitor therapy. The neutrophils conferred poor survival for PDACs. Increasing evidence suggests that tumors manipulate neutrophils, sometimes in the initial stage of their differentiation process, to create a variety of phenotypic and functional polarization states that have impact on tumor behavior (43). Neutrophils are an essential component of inflammation process, and inflammation plays a critical role in initiating tumorigenesis. Neutrophils can promote tumor growth *via* the induction of angiogenesis and actively participate in the process of the metastatic cascade (43). Our study also suggests that the resting of dendritic cells (DCs) is associated with favorable OS and CR. DCs can promote immune evasion of tumor cells by presenting tissue-specific antigens to regulatory T cells that leads to tumor-specific immunosuppression. Jang et al. reported that the interaction network between Treg cells and tumor-associated DCs can suppress the expression of costimulatory ligands necessary for the effector CD8+ T cell response (44). Argentiero et al. showed that activated DCs in PDAC patients with metastatic lymph nodes may upregulate the WNT pathway that participates in the immunosuppressive process (45). Up to now, although the function of tumor-infiltrating B cells in PDAC has not been extensively investigated, there are compelling findings for the involvement of B cells in inducing and processing pancreatic tumorigenesis. Mechanisms of this function include suppressor of other tumor-infiltrating antitumor immune cells such as CD8 + T cells in the tumor microenvironment and promoting cancer cell proliferation (46). However, we found naïve B cells are associated with good OS. This contradictory finding needs further exploration. We found that natural killer cells, well-known to exhibit antitumor activity, were associated with worse RFS in univariate analysis. However, log rank test (p=0.3) indicated that this association might be a false positive.

Differences existed in molecular subtypes regarding the relevance between immune cell fractions and clinical outcomes. Within the classical subtype, estimated high levels of plasma cells, naïve B cells, and low fractions of M0 macrophages and neutrophils were associated with improved OS in PDACs. Plasma cells also associated with good RFS. Previously studies reported plasma cells to be prognostic across solid tumors (10, 11, 47). Among the basal-like subtypes, the high levels of M0 macrophages and activated mast cells were associated with poor OS. In addition, the M2 macrophages, known to play an immune-suppressive protumorogenic role, conferred worse RFS. Focal adhesion kinases inhibition increases immune surveillance and response to immune checkpoint blockade *via* overcoming the tumor fibrosis and reduces the number of M2 macrophages of PDAC (48).

The consensus clustering analysis with immune cell fractions demonstrated six distinctive immunologic subtypes of PDAC with prognostic implications. And the immune subgroups were significantly associated with tumor stage, metastasis, grade, and molecular subtypes. The underlying molecular mechanism driving the establishment and maintenance of PDAC immune phenotypes deserves to be addressed in integrative analysis with multi-omics data in our upcoming studies.

After antigen recognition, PD-1 is expressed on the surface of active T-cells, and PDL-1 and PD-L2 are ligands for the PD-1 receptor on T-cells. Higher levels of PD-1/immune content score are associated with favorable OS. In contrast, the higher levels of PDL-1 and PDL-2 were found to be associated with poor OS. ICIs targeting the PD-1/PD-L1 axis in combination with other kinds of immunotherapy or chemoradiotherapy are under investigation in PDAC (49).

There are inevitable limitations in our study. The primary one is that the immune content and fractions of cell subsets were computationally estimated. In a more ideal situation, information should be measured with direct experimental method. We are reassured, as our finding regarding the immune cell type is consistent with the publications based on histologically characterized cohorts. The second one is the lack of proportion of clinical information, such as metastasis and grade, decreasing the statistical power of the multivariable models. Finally, the findings reported here need to be validated in further biological experiments and independent clinical cohorts.

To conclude, as an unbiased in silico computationally study with a large pooled cohort, our work demonstrates the complex immune landscape in PDAC. We showed that the fractions of M0 macrophages, neutrophils, and CD8+ T cells are associated with clinical outcomes of PDACs. In addition, we observed the expression of immune checkpoint PD-1 is associated with survival of PDACs. Our work has clinical translational value for the availability and increasing usage of immunotherapies in PDAC. These findings suggest potential novel therapeutic target and drug combination strategies that merit further investigation.

## Data Availability Statement

Publicly available datasets were analyzed in this study. This data can be found here: TCGA (PAAD), ICGC (PACA-CA, PACA-AU), ArrayExpress (E-MTAB-6134), Gene Expression Omnibus (accession numbers: GSE71729, GSE17891, GSE79668,GSE57495, GSE78229, GSE85916).

## Author Contributions

RL contributed to the study design, performed statistical analysis and interpretation, and drafted the manuscript. Y-ZL contributed to the data collection. All authors contributed to critical revision of the final manuscript. RL approved the final version of the manuscript. All authors contributed to the article and approved the submitted version.

## Funding

This study was supported by the National Scientiﬁc Foundation of China (No. 31801121) and the National Natural Science Foundation of Hunan (No. 2020JJ5879).

## Conflict of Interest

The authors declare that the research was conducted in the absence of any commercial or financial relationships that could be construed as a potential conflict of interest.

## Abbreviations

PDAC, Pancreatic Ductal Adenocarcinoma; OS, overall survival; RFS, relapse-free survival; CIBERSORT, Cell type identification by estimating relative subset of known RNA transcripts; GEO, Gene Expression Omnibus; SVM, support vector machine; SD, standard deviation; HR, hazard ratio; CI, confidence interval; OR, odds ratio; CR, complete response; ICGC, International Cancer Genome Consortium; TCGA, The Cancer Genome Atlas; FPKM, fragments per kilobase of transcript per million; TPM, transcripts per kilobase million; DFS, disease-free survival; Tregs, T cells regulatory; DCs, dendritic cells; ICI, immune checkpoint inhibitor; CI, confidence interval.
