# Supplementary material for: Relevance of Immune Infiltration and Clinical Outcomes in Pancreatic Ductal Adenocarcinoma Subtypes
Source: Front Oncol. 2021 Jan 6;10:575264. doi: 10.3389/fonc.2020.575264 (PMC7815939; doi:10.3389/fonc.2020.575264)
Supplement: Supplementary file 2 [file DataSheet_1.docx]

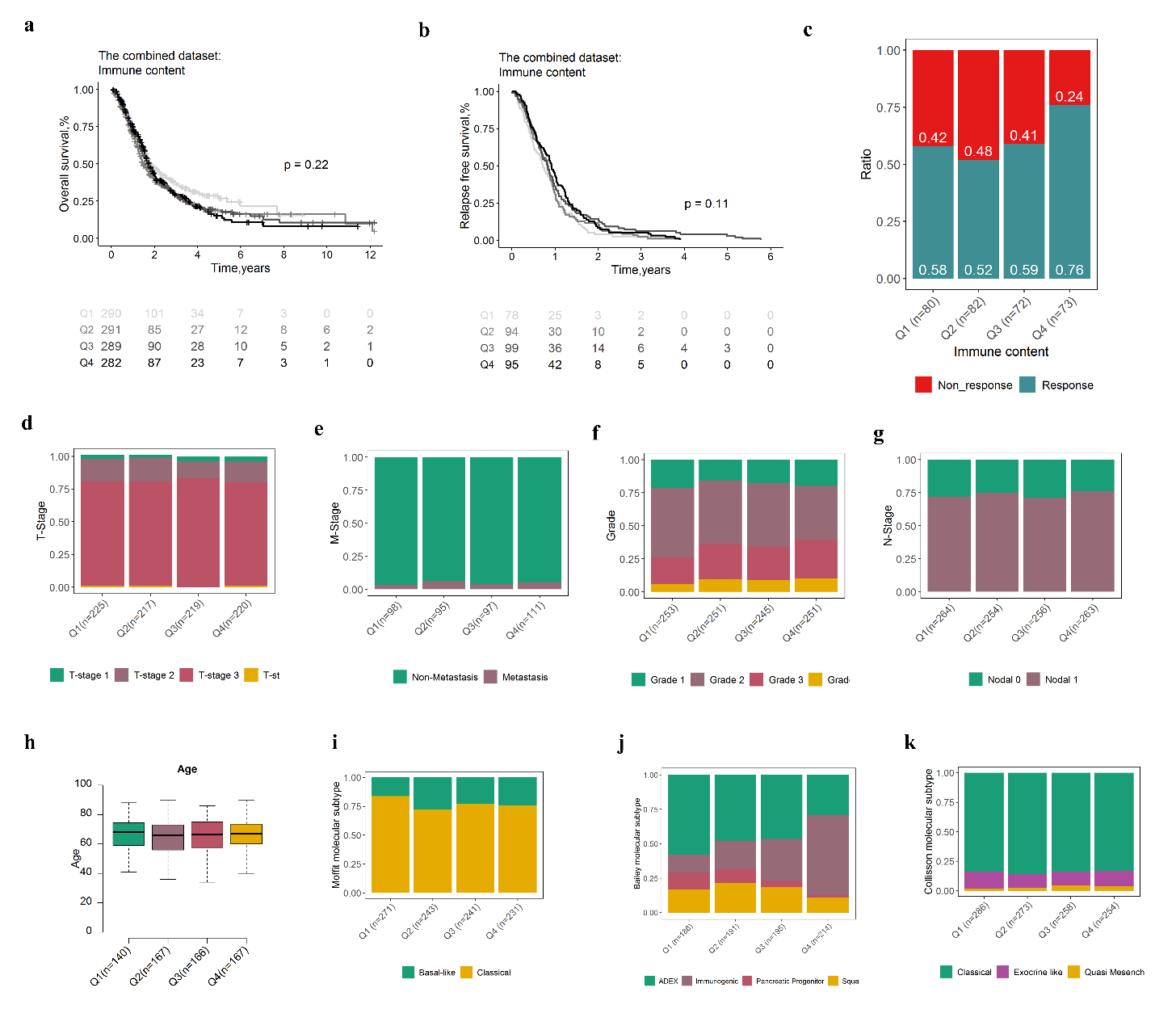
**Supplementary figure 1**: **Associations between immune content score and clinical covariates**. Survival plots for overall survival (a) and relapse free survival (b) by quantiles of immune content score. P-values from log-rank tests are shown. Spine plots for complete response by quantiles of immune content score (c). Spine plots of the relationship between immune content score and tumour size (d), metastasis (e), grade (f), nodal (g). box plots of the distribution of age at diagnosis by immune content score (h). Spine plots of the relationship between immune content score and Moffit subtype (i), Bailey subtype (j) and Collision subtype (k).

**
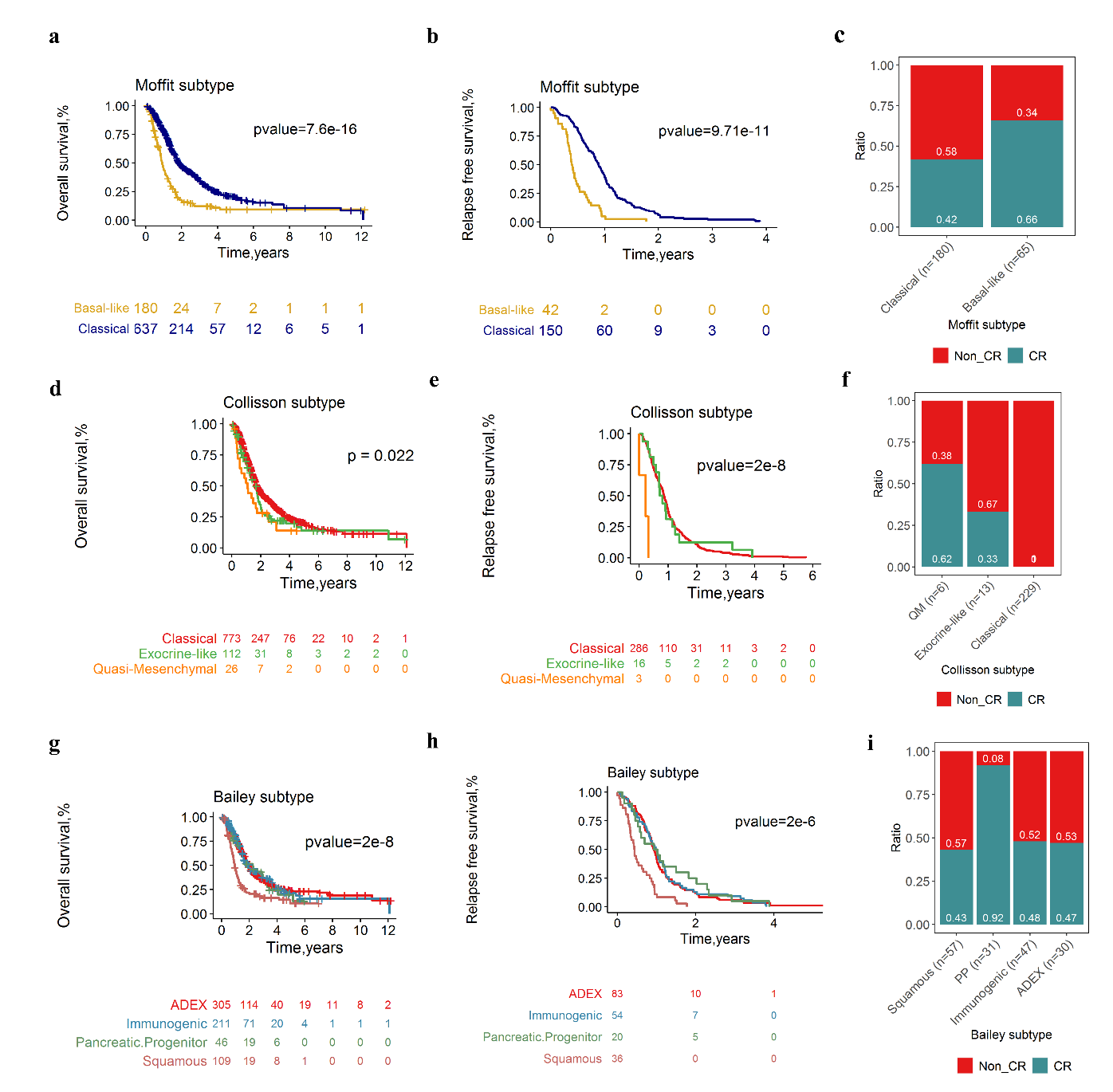
**

**Supplementary Figure 2**. Survival plots for overall survival and relapse free survival and spine plots for complete response by Moffit (a-c), Collisson (d-f) and Bailey (g-i) molecular subtypes. P-values from log-rank tests are depicted. Abbreviations: Non-CR: non-complete response, CR: complete response.


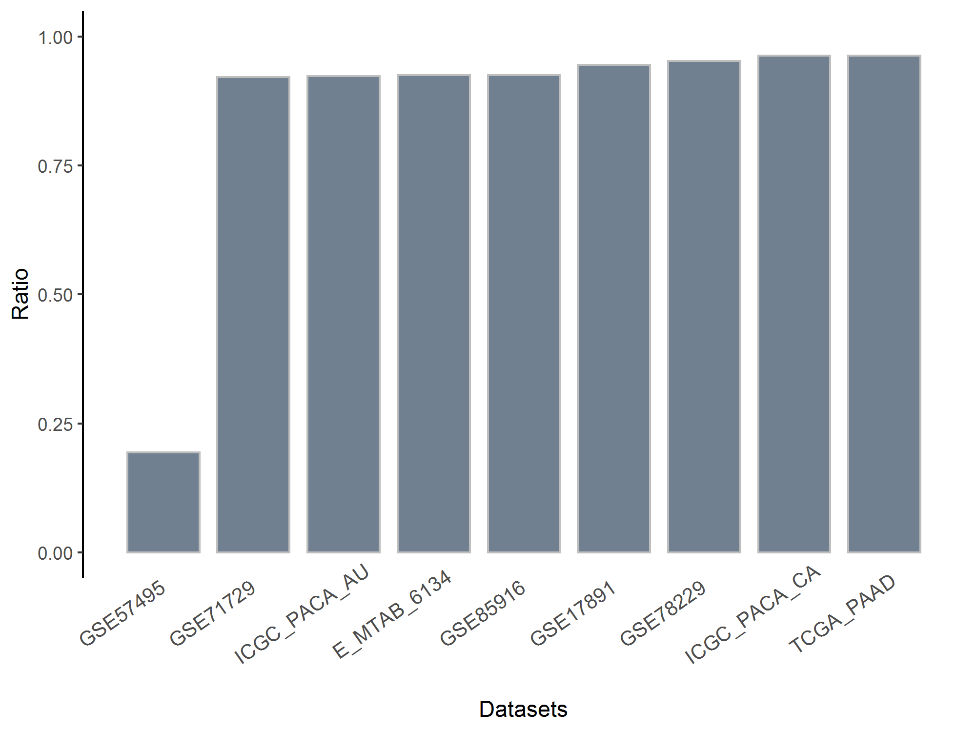


**Supplementary figure 3**: Bar plot of the proportion of the LM22 547 signature matrix genes available by datasets.


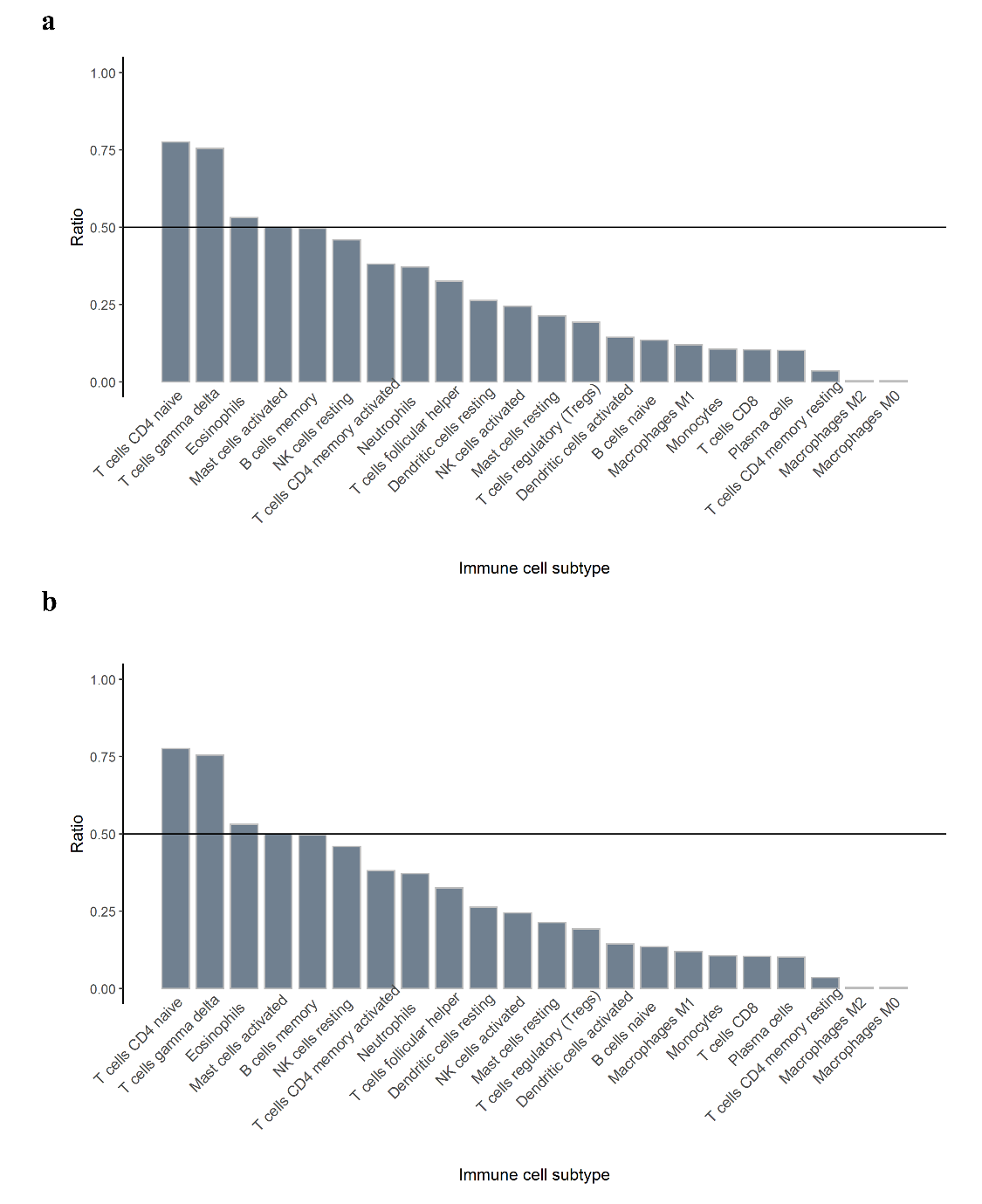


**Supplementary figure 4**: Bar plot of the overall proportion of the samples have no relative (a) and absolute (b) infiltrations of individual immune cell subsets in the whole dataset.


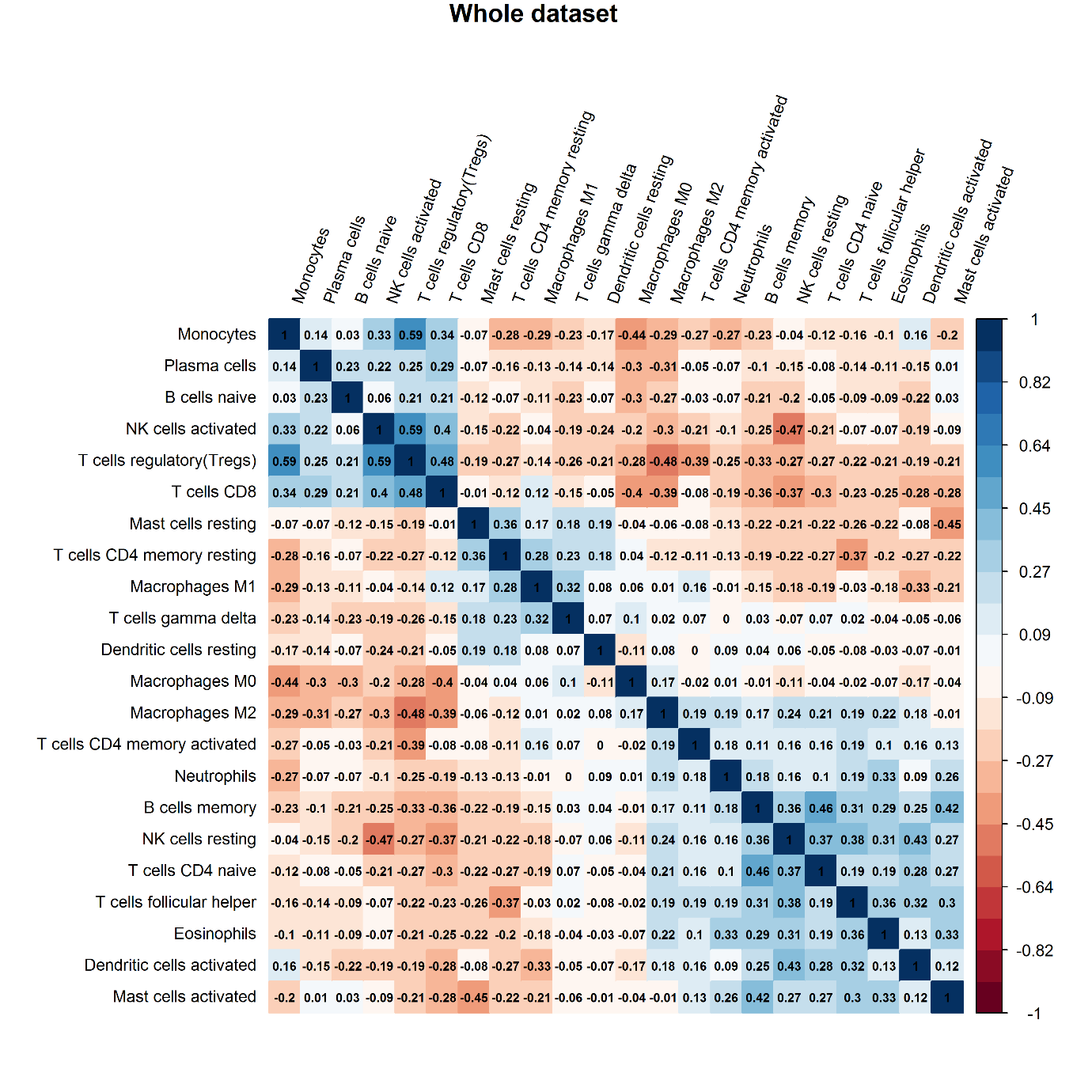
**Supplementary figure 5**. Correlation matrix of all 22 immune cell proportions in the whole dataset.


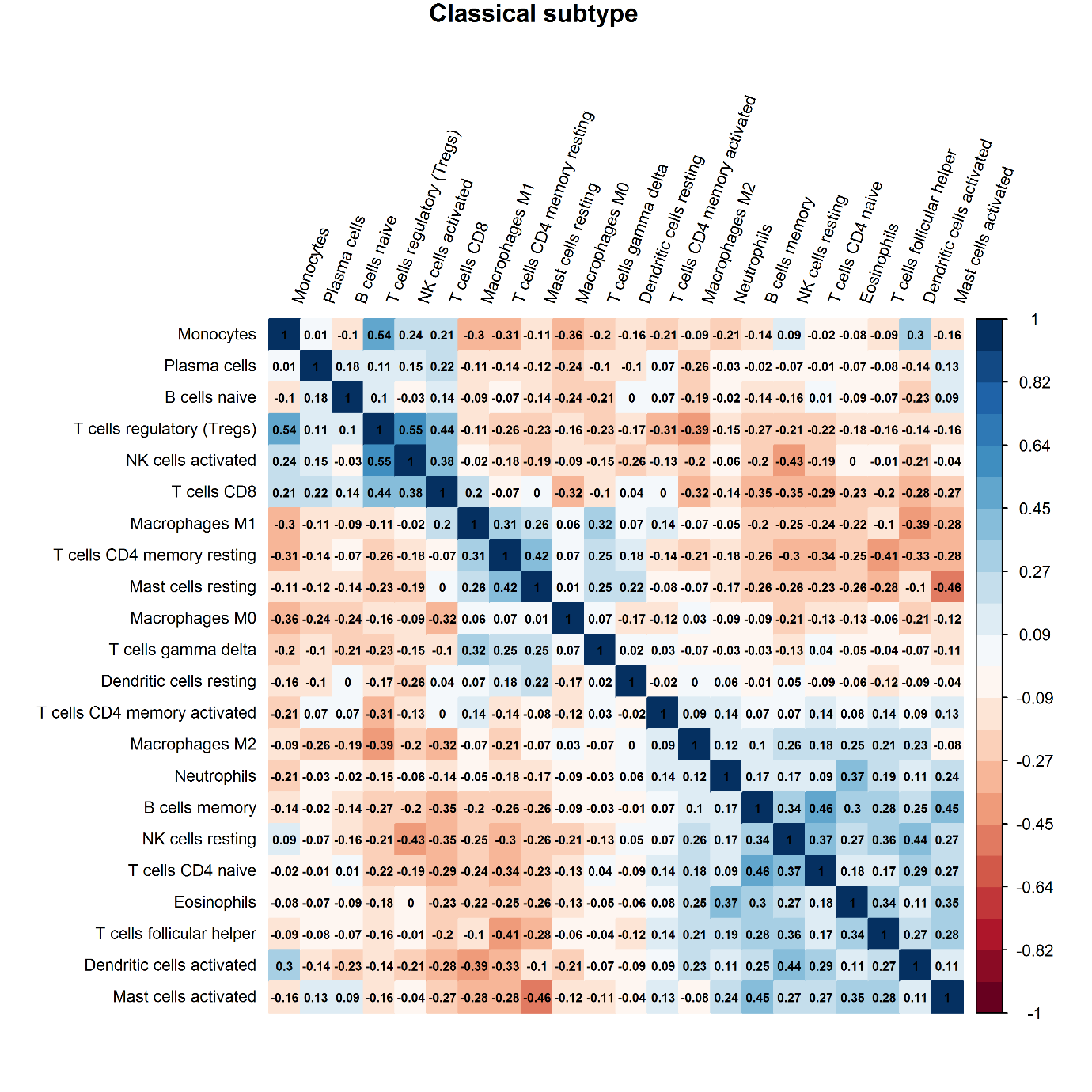


**Supplementary figure 6**. Correlation matrix of all 22 immune cell proportions in the classical subtype.


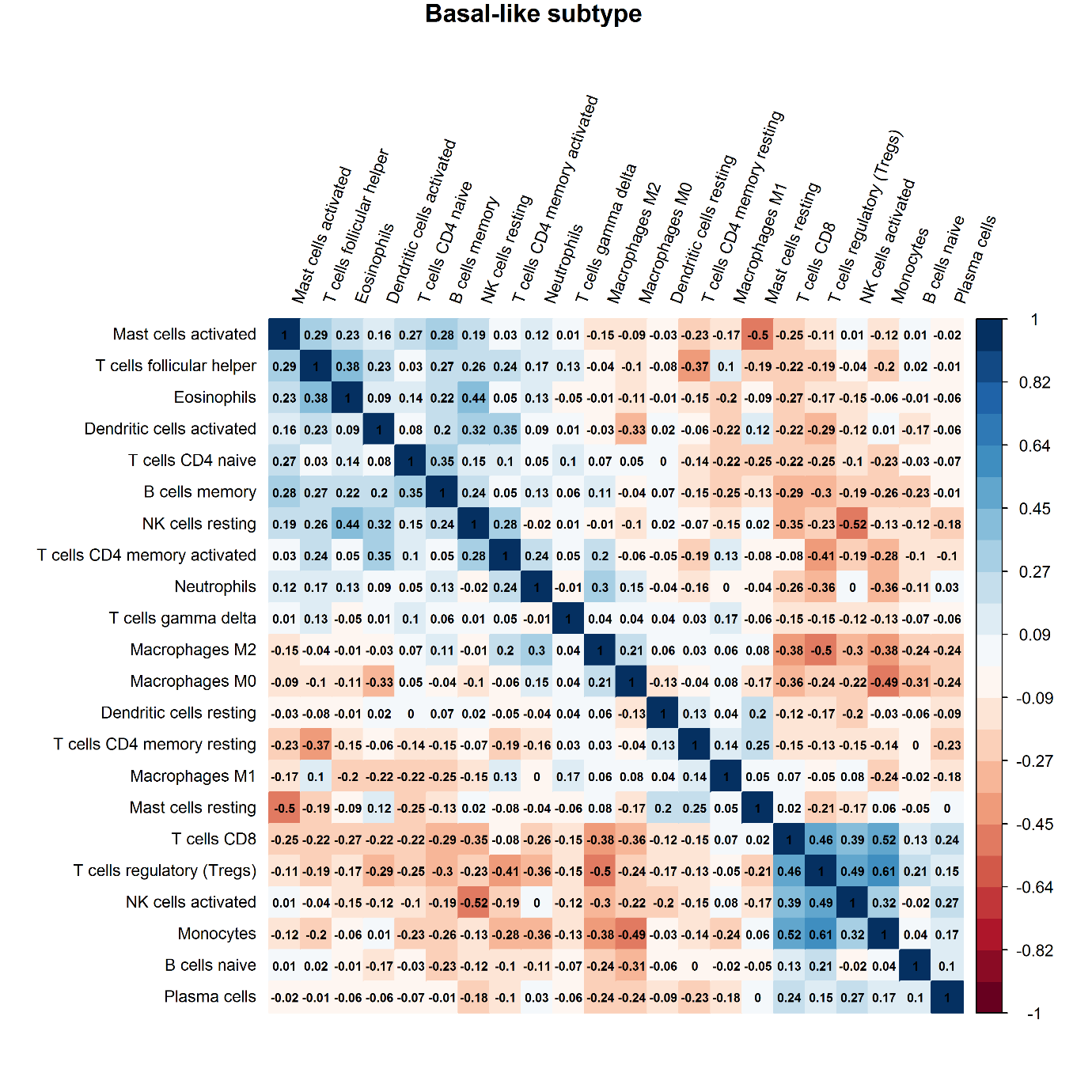
**Supplementary figure 7.** Correlation matrix of all 22 immune cell proportions in the basal-like subtype.


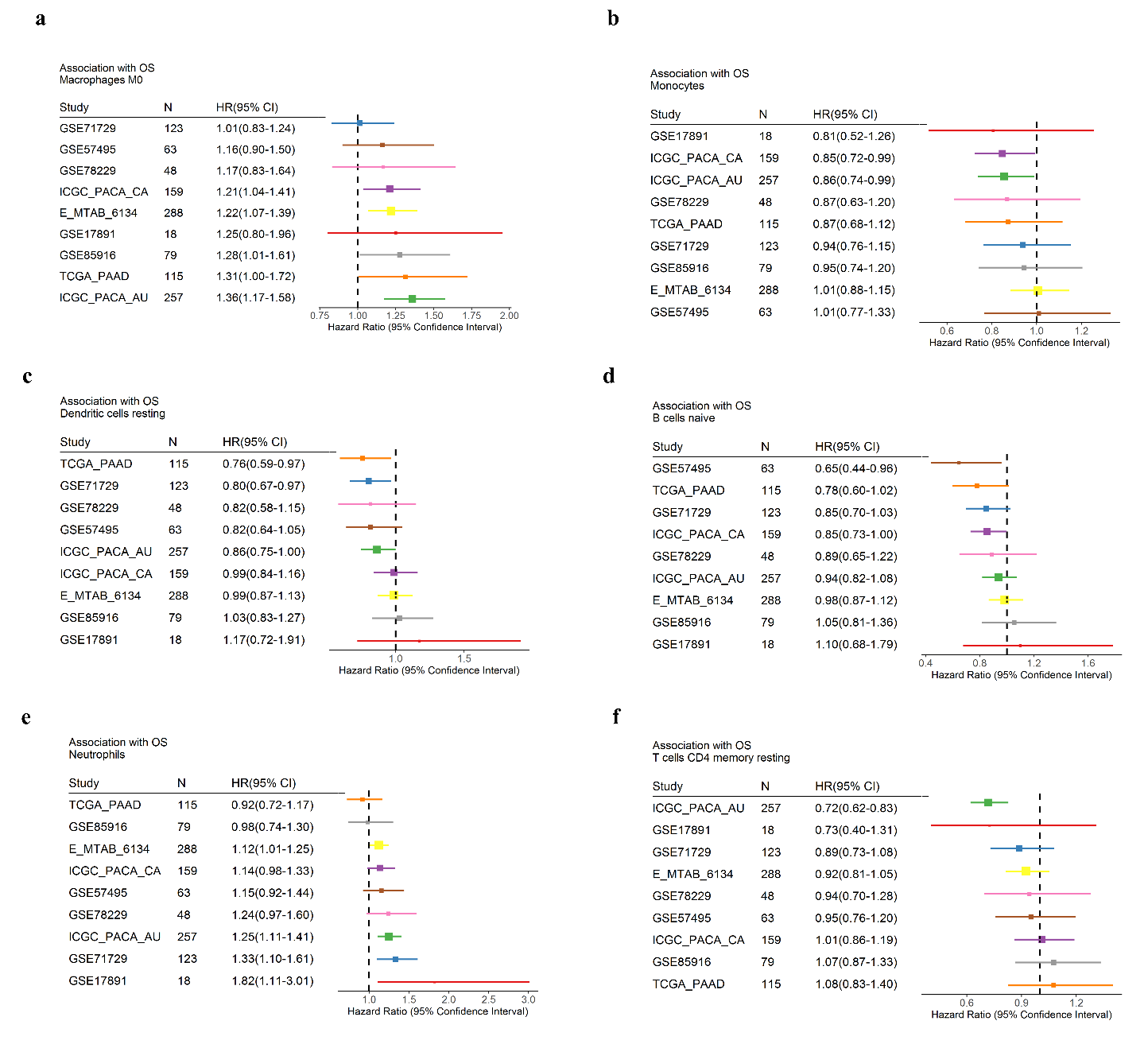


**Supplementary figure 8.** Forest plots showing the HRs (and 95% CI) of the M0 macrophages (a), monocytes (b), resting dendritic cells (c), naïve B cells (d), neutrophils (e) and resting CD4+ T cells (f) for overall survival across datasets utilized in this study.

**
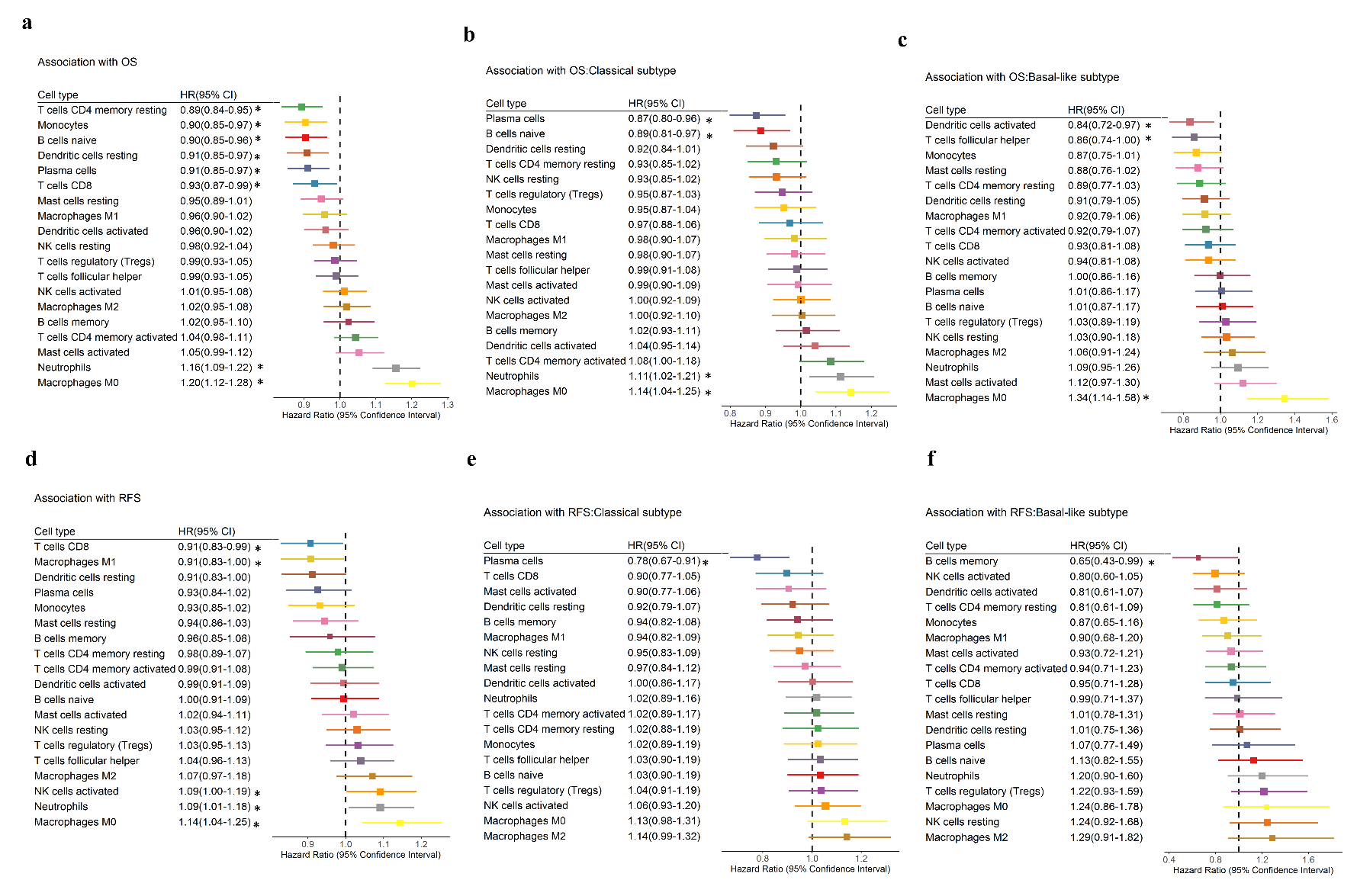
**

**Figure S9. Associations between overall survival, relapse-free survival and immune cells overall and within Moffit subtypes.** Unadjusted HRs (boxes) and corresponding 95% confidence intervals (horizontal lines) for immune cell subtypes (estimated in absolute mode) associated with overall survival (a-c) and relapse free survival (d-f). Box size is inversely proportional to the standard error of HR. * indicate HRs with a p value < 0.05. Immune cell fractions are stratified as quartiles for the survival curves (c–h), and p-values be calculated using log-rank tests were drawn. HR, hazard ratio.


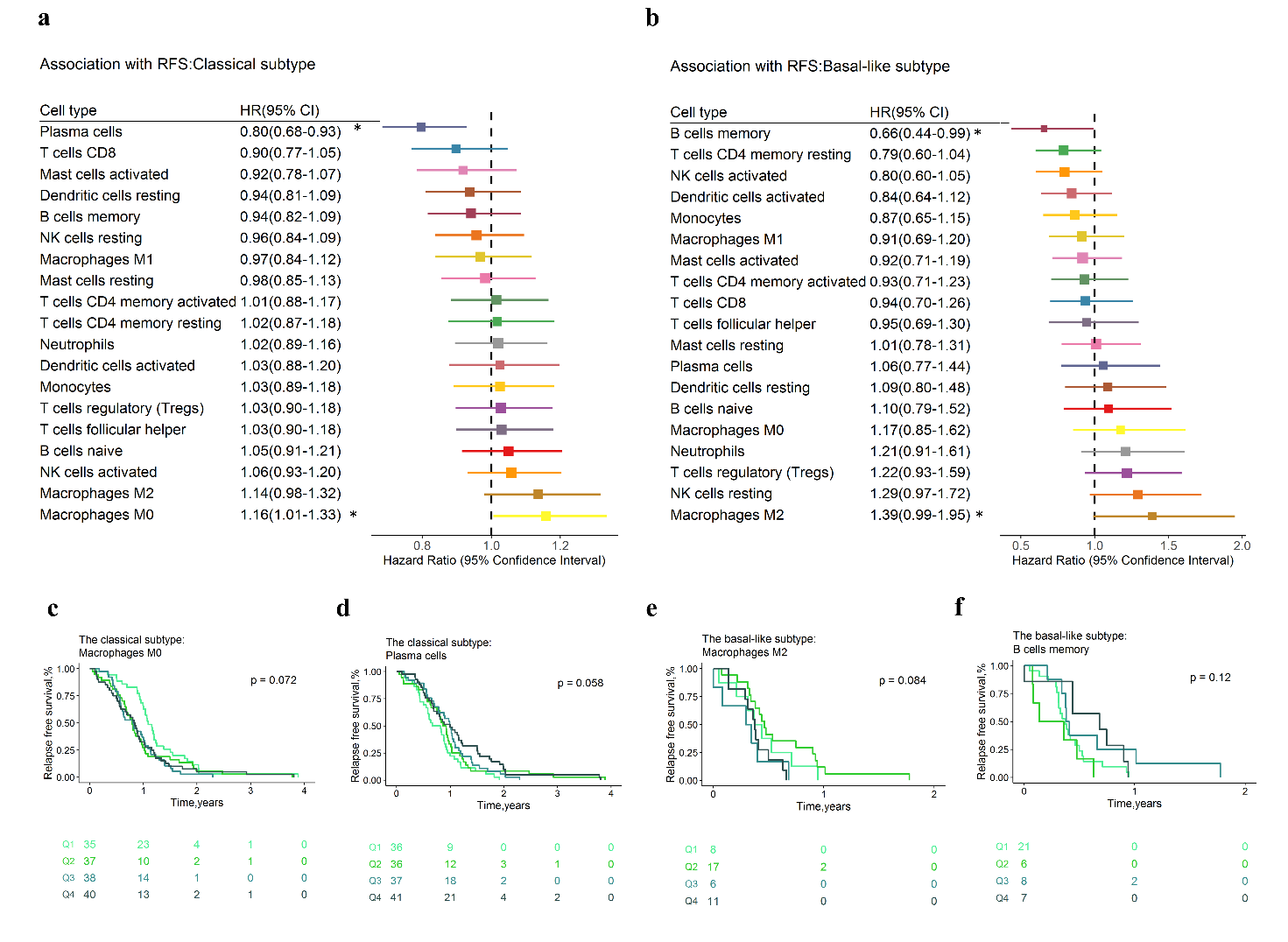


**Supplementary figure 10. Associations between relapse-free survival and immune cells by Moffit molecular subtype.** Unadjusted HRs (boxes) and 95% confidence intervals (horizontal lines) for the association with relapse-free survival in Moffit molecular subtypes (a–b). Box size is inversely proportional to the standard error of HR. * indicates a p-value < 0.05. In the survival curves, immune cell subsets are stratified as quartiles (c–f). P-values from log-rank tests are shown. HR, hazard ratio.


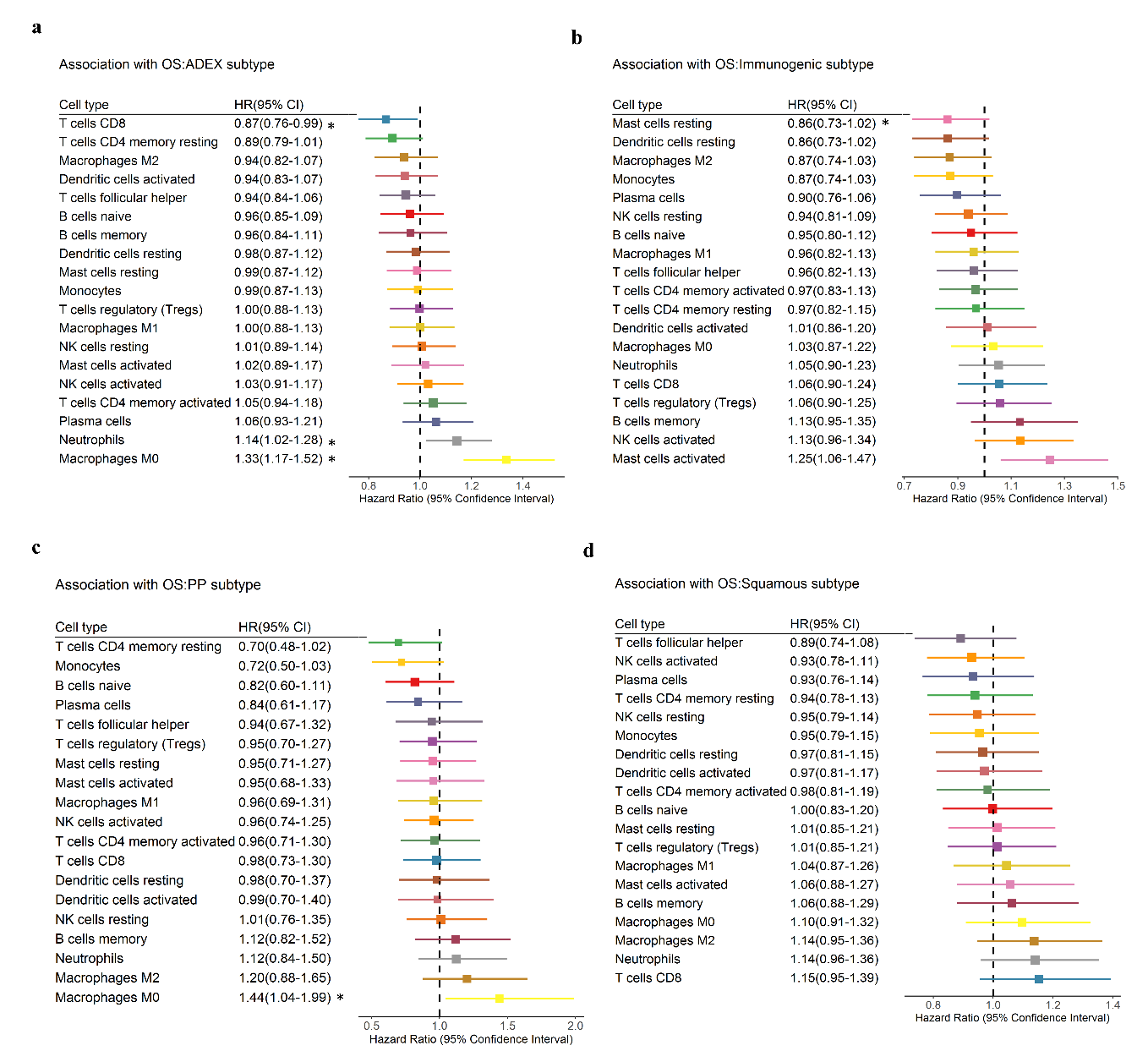


**Supplementary figure 11. Associations between overall survival and immune cells by Bailey molecular subtype.** Unadjusted HRs (boxes) and 95% confidence intervals (horizontal lines) for the association with overall survival in Bailey molecular subtypes (a–b). Box size is inversely proportional to the standard error of HR.* denote a p-value < 0.05. HR, hazard ratio.


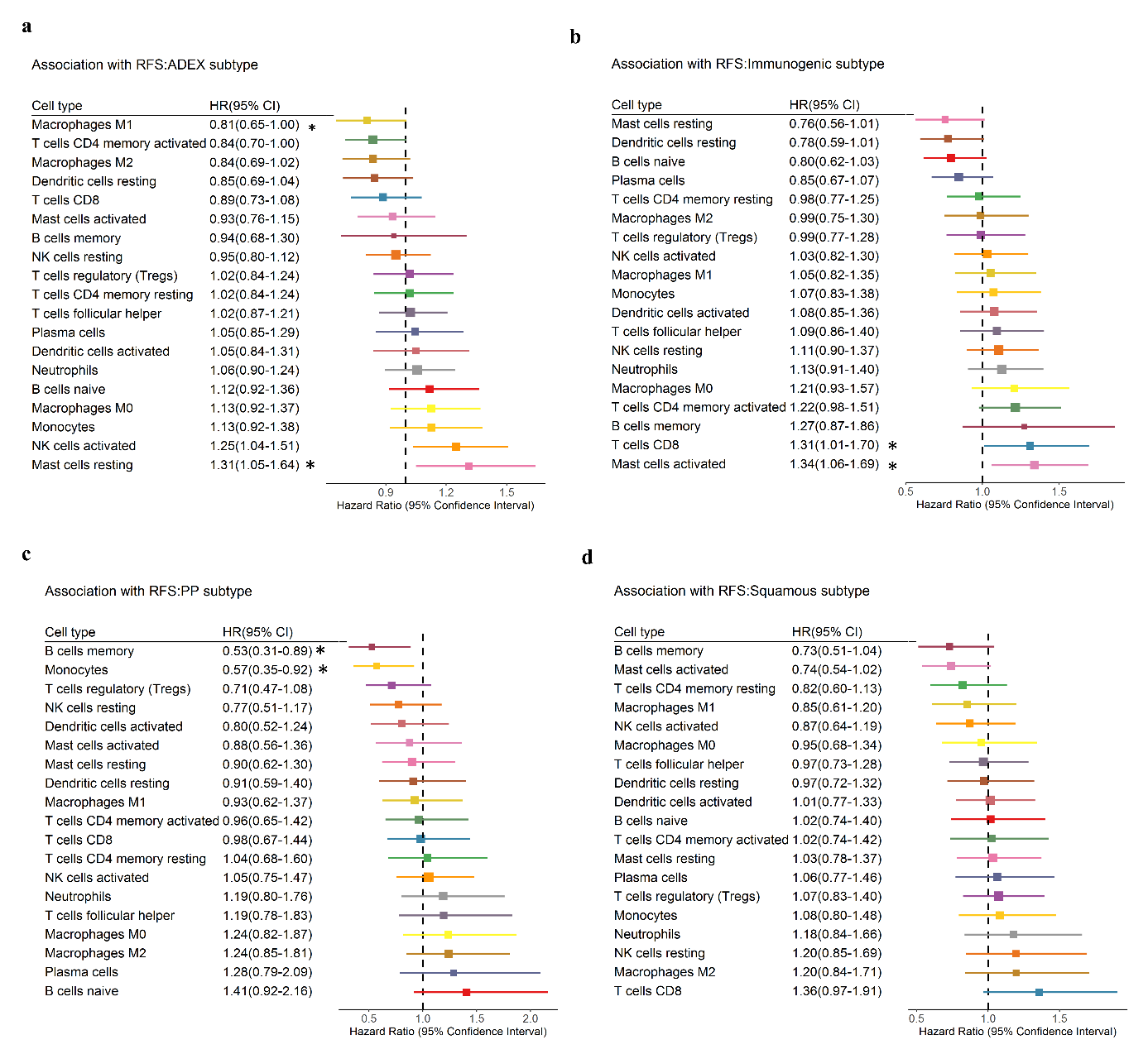


**Supplementary figure 12. Associations between relapse-free survival and immune cells by Bailey molecular subtype.** Unadjusted HRs (boxes) and 95% confidence intervals (horizontal lines) for the association with relapse-free survival in Bailey molecular subtypes (a–b). Box size is inversely proportional to the standard error of HR. * denote a p-value < 0.05. HR, hazard ratio.


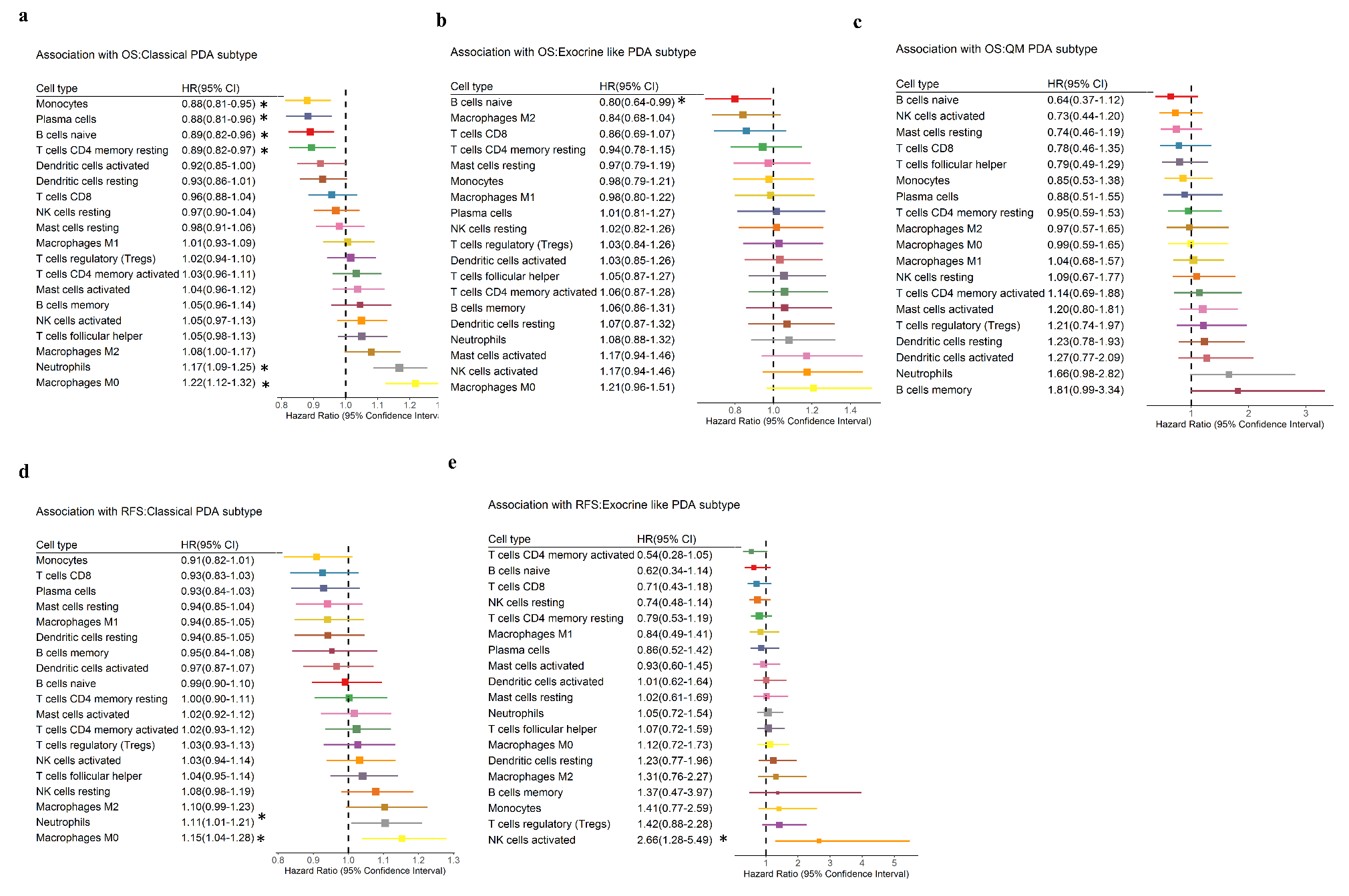


**Supplementary figure 13.** Associations between immune cells and survival by Collisson molecular subtype. Unadjusted HRs (boxes) and 95% confidence intervals (horizontal lines) for the association with overall survival (a-c) and relapse-free survival (d-e) in Collisson molecular subtypes. Box size is inversely proportional to the standard error of HR. * denote a p-value < 0.05. HR, hazard ratio.


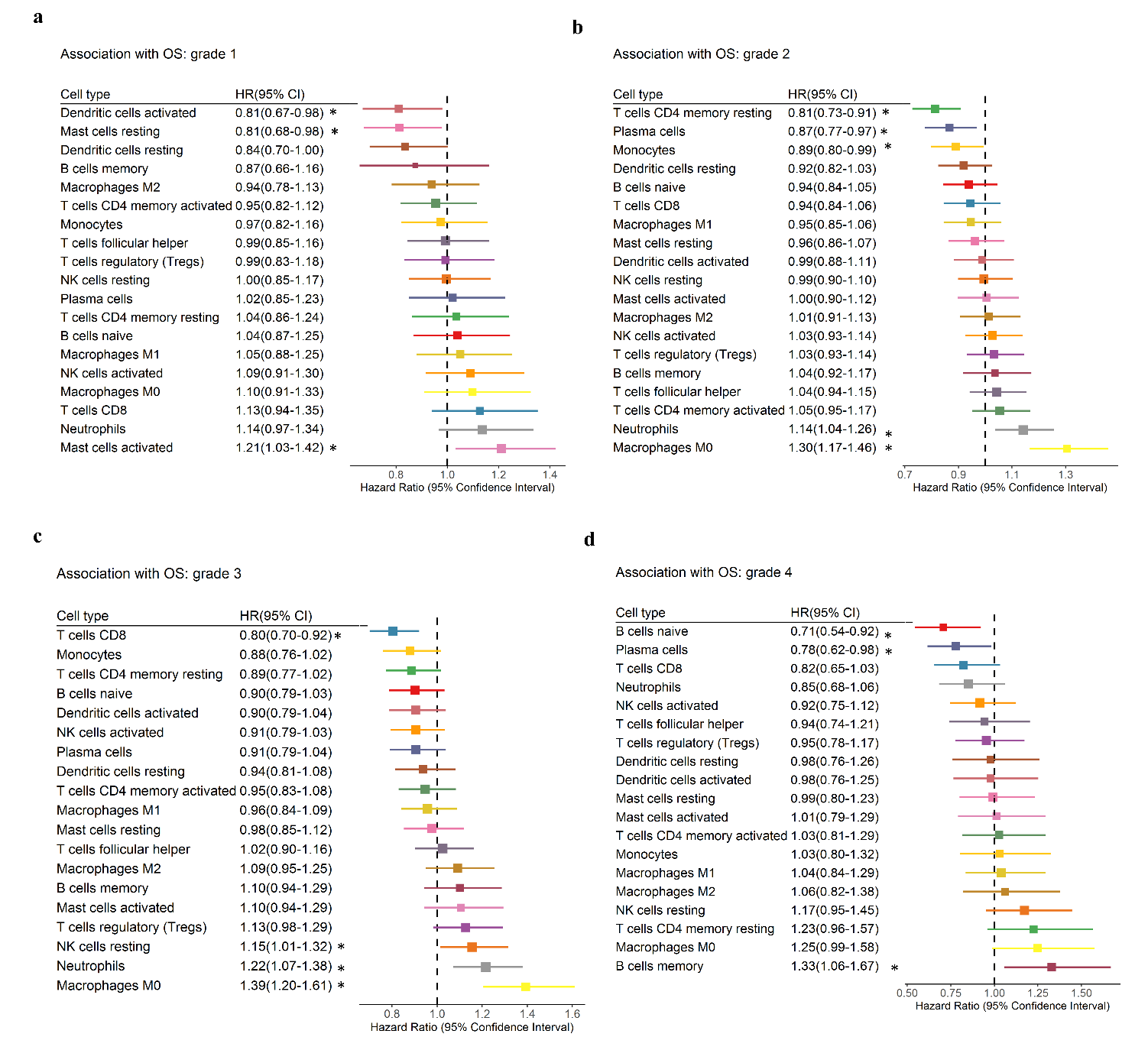


**Supplementary figure 14.** Associations between immune cells and overall survival by group. Unadjusted HRs (boxes) and 95% confidence intervals (horizontal lines) for the association with overall survival in different groups. Box size is inversely proportional to the standard error of HR. * denote a p-value < 0.05. HR, hazard ratio.


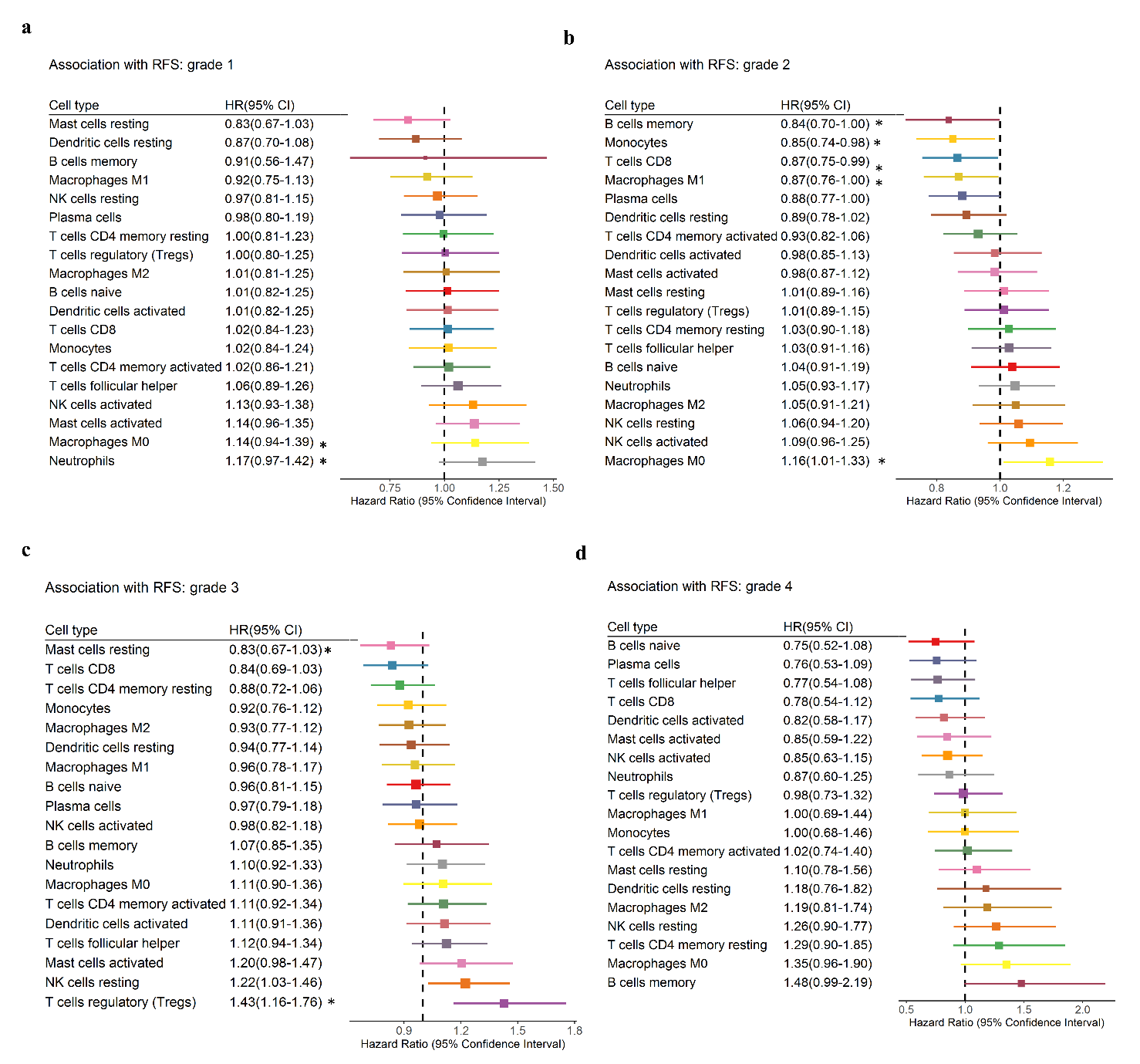


**Supplementary figure 15.** Associations between immune cells and relapse-free survival by group. Unadjusted HRs (boxes) and 95% confidence intervals (horizontal lines) for the association with relapse-free survival in different groups. Box size is inversely proportional to the standard error of HR. * denote a p-value < 0.05. HR, hazard ratio.


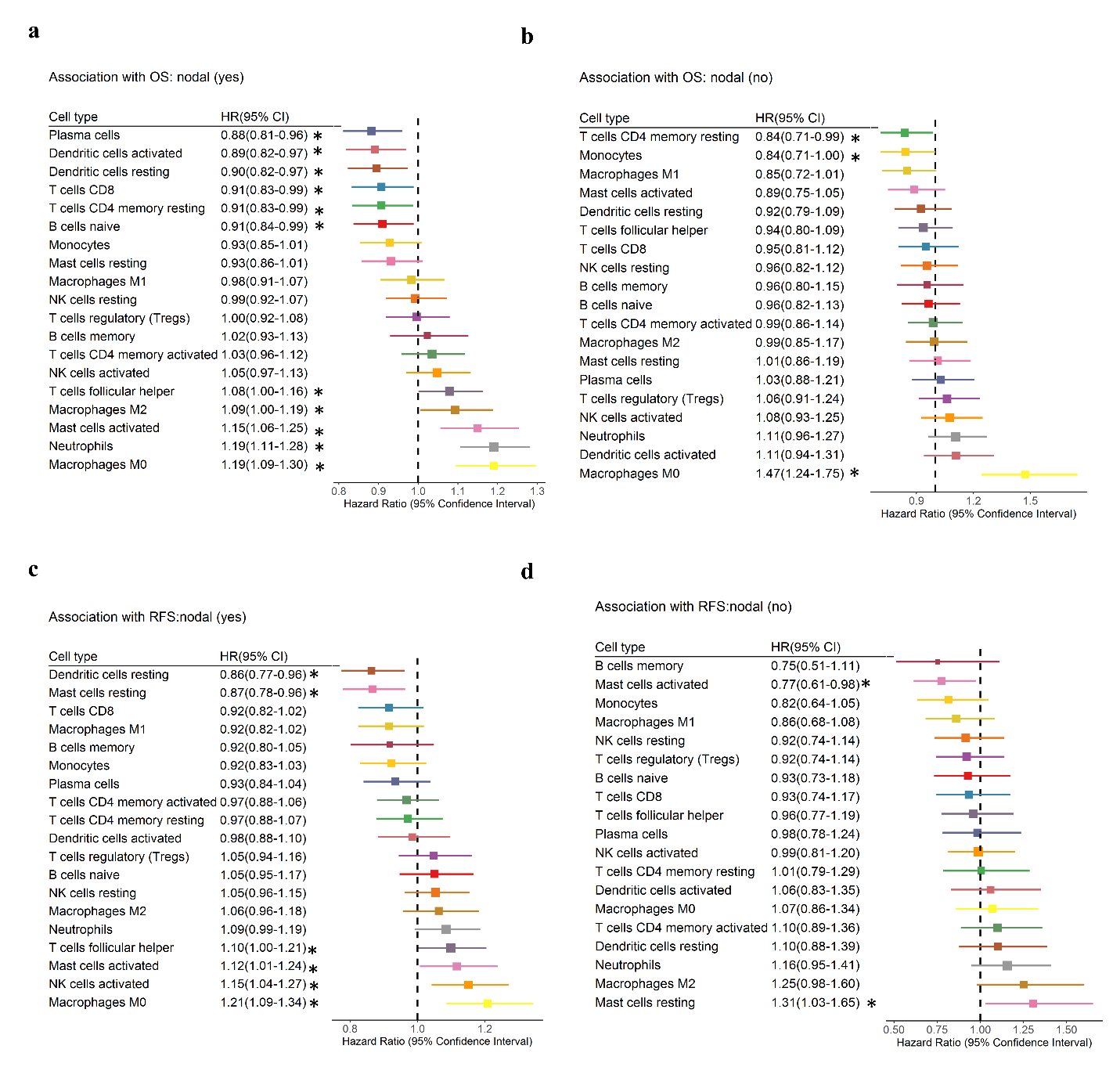


**Supplementary figure 16.** Associations between immune cells and survival by nodal status. Unadjusted HRs (boxes) and 95% confidence intervals (horizontal lines) for the association with overall survival (a-b) and relapse-free survival (c-d) in positive and negative nodal status. Box size is inversely proportional to the standard error of HR. * denote a p-value < 0.05. HR, hazard ratio.


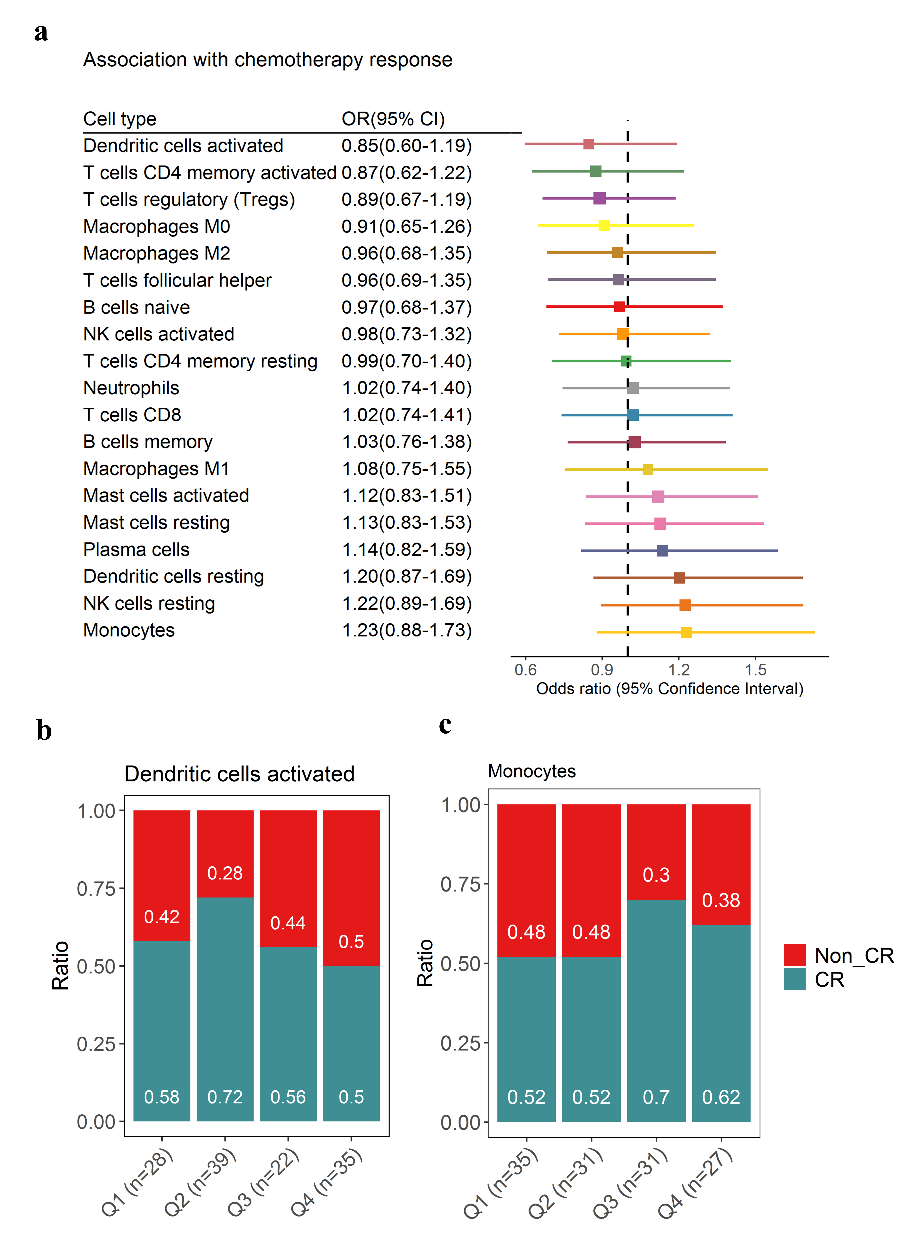


**Figure 17. Associations between complete response and tumour infiltrating immune cell subtypes overall.** Unadjusted ORs (boxes) and 95% CIs (horizontal lines) for the association with complete response (a). Box size is inversely proportional to the standard error. Spine plots illustrating the distribution of complete response rates within quartiles of tumour infiltrating immune cell subtypes (b-d). CI: confidence interval.


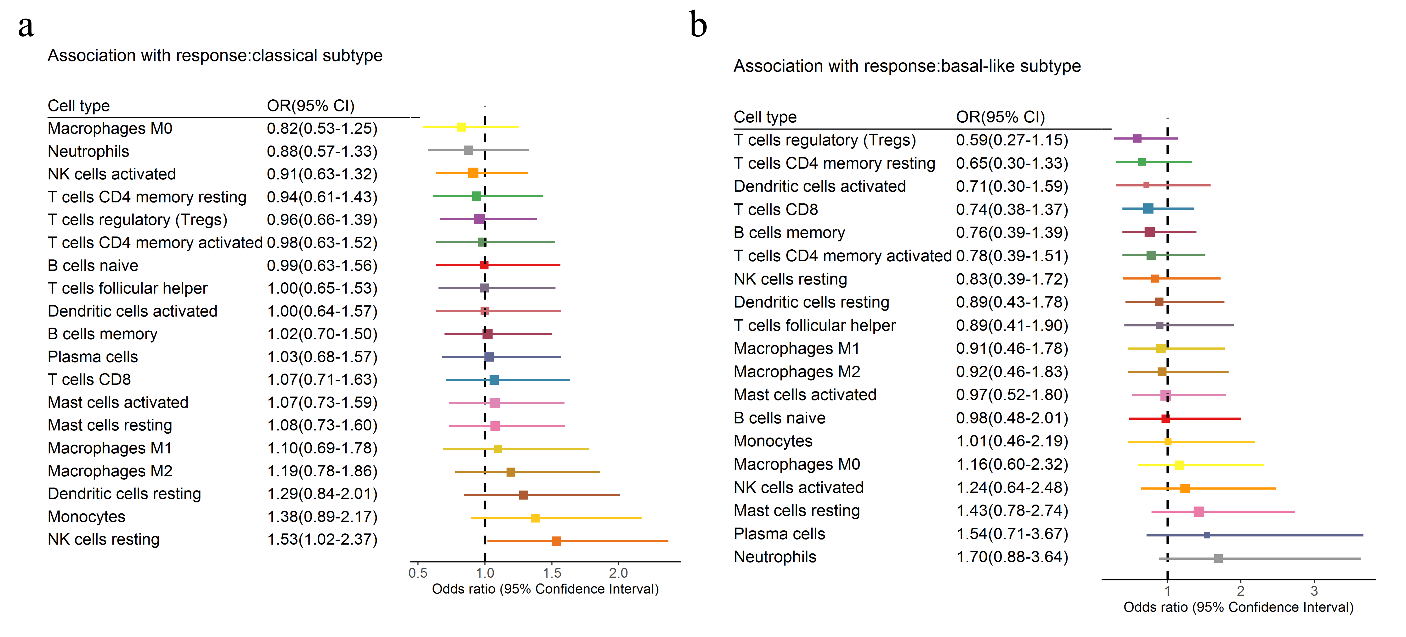


**Supplementary figure 18.** Forest plots showing the ORs (and 95% CI) of the univariate logistic analyses for complete response to chemotherapy within classical (a) and basal-like subtype (b). OR: odds ratio.


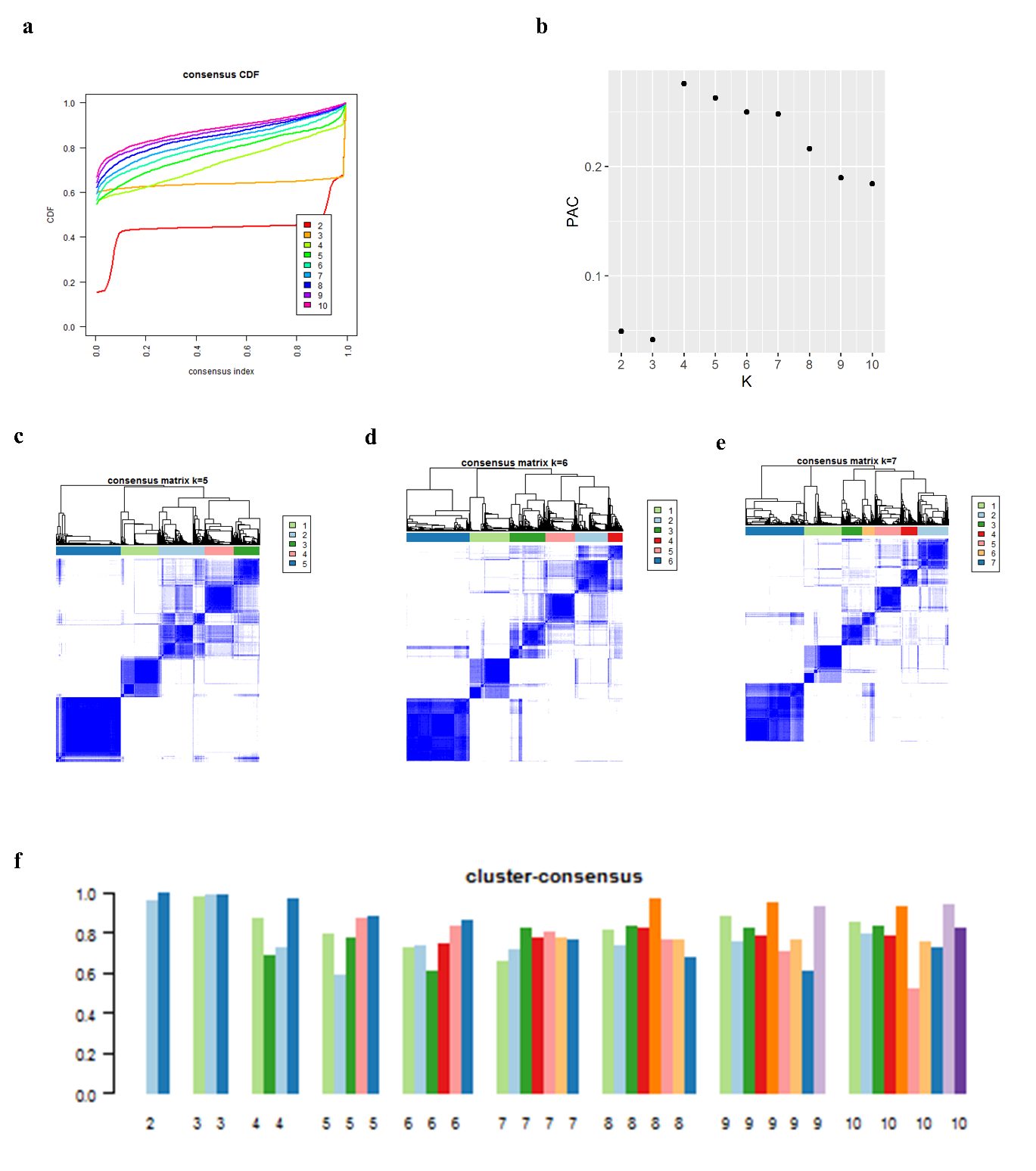
**Supplementary figure 19**. The cumulative distribution function (a) demonstrated that low k values at 3,4,5 underfit the dataset whereas k=8,9,10 overfits the data. To discern whether k=5 or k=6 or k=7 better fit our dataset, we calculated the proportion of ambiguous clusters values. k=5 had a lower proportion of ambiguous clusters value compared to k=4 (b). Both the consensus matrix (c-e) and consensus cluster index (number of times cluster stays consistent; f) also supported that k=6 was more stable than k=5 or k=7.


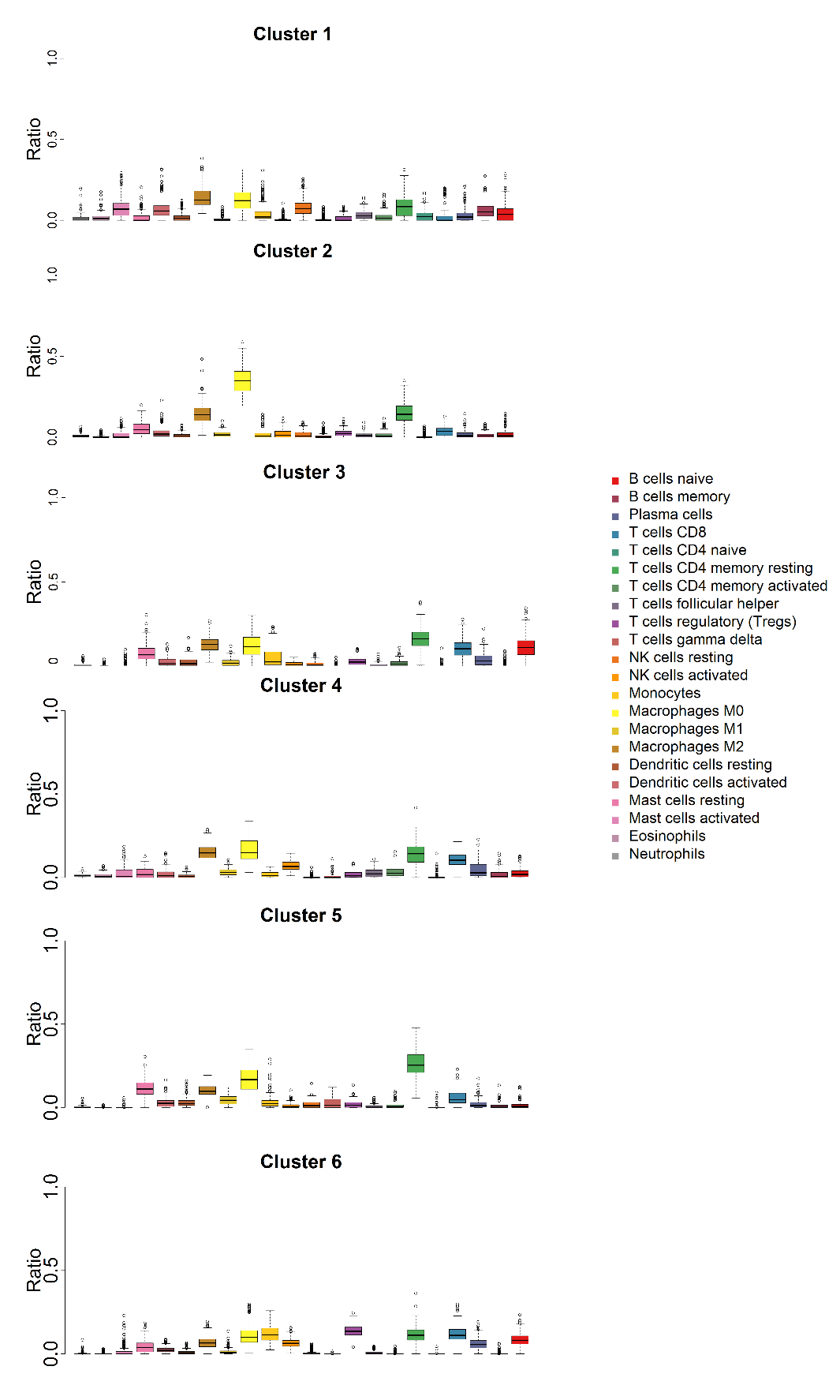


**Supplementary figure 20.** Box plots depicting the distribution of each immune cell type across six immune clusters.

###
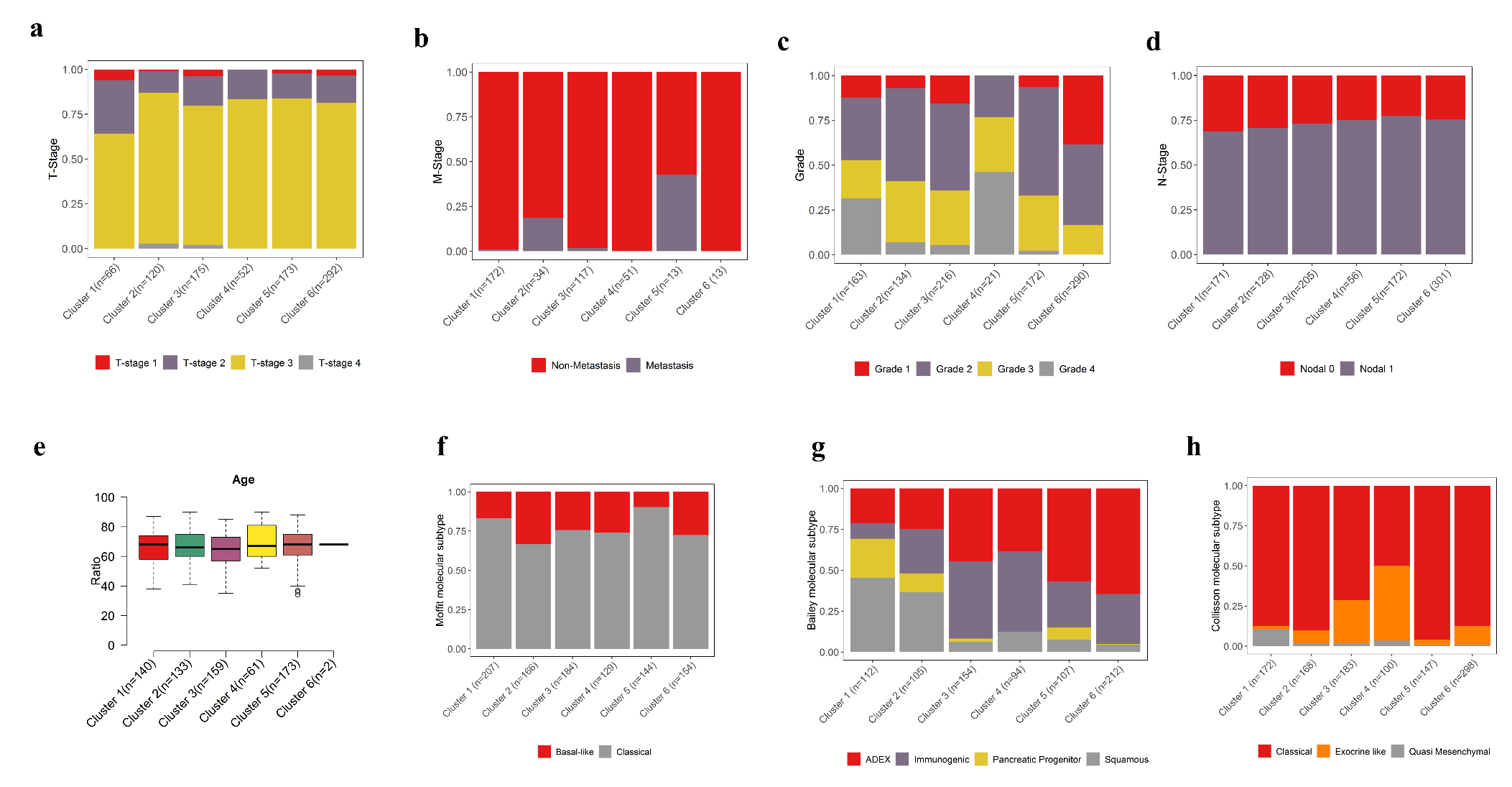


### Supplementary figure 21. Associations between immune cell cluster and clinical covariates. Spine plots of the relationship between immune content score and tumour size (a), metastasis (b), grade (c), nodal (d). Box plots of the distribution of age at diagnosis by immune content score (e). Spine plots of the relationship between immune content score and Moffit subtype (f), Bailey subtype (g) and Collision subtype (h).
